# Current trends and challenges in the clinical use of cardiovascular magnetic resonance: a survey from the Italian Society of Cardiology

**DOI:** 10.1093/ehjimp/qyaf046

**Published:** 2025-04-17

**Authors:** Lorenzo Monti, Fabrizio Ricci, Andrea Baggiano, Andrea Barison, Nazario Carrabba, Stefano Figliozzi, Patrizia Pedrotti, Camilla Torlasco, Erika Tempo, Alessandro Giaj Levra, Stefania Paolillo, Gianfranco Sinagra, Pasquale Perrone Filardi, Ciro Indolfi, Santo Dellegrottaglie

**Affiliations:** Istituti Clinici Scientifici Maugeri IRCCS, Radiology Department, Via Maugeri 10, 27100 Pavia, Italy; Department of Neuroscience, Imaging and Clinical Sciences, G. D’Annunzio University of Chieti-Pescara, 66100 Chieti, Italy; University Cardiology Division, SS Annunziata Polyclinic University Hospital, 66100 Chieti, Italy; Department of Cardiovascular Imaging, Centro Cardiologico Monzino IRCCS, 20138 Milano, Italy; Department of Cardiology, Fondazione Toscana Gabriele Monasterio, 56127 Pisa, Italy; Cardio-Thoraco-Vascular Department, Careggi Hospital, 50134 Florence, Italy; Cardio Center, IRCCS Humanitas Research Hospital, 20089 Rozzano, Milano, Italy; Department of Biomedical Sciences, Humanitas University, 20072 Pieve Emanuele, Milano, Italy; S.S. Cardiologia Diagnostica per Immagini—RM Cardiaca; S.C. Cardiologia 4 Diagnostica-Riabilitativa Dipartimento CardioToracoVascolare ‘De Gasperis’, ASST Grande Ospedale Metropolitano Niguarda, 20162 Milano, Italy; Department of Cardiology, Istituto Auxologico Italiano, IRCCS, 20149 Milano, Italy; Istituti Clinici Scientifici Maugeri IRCCS, Radiology Department, Via Maugeri 10, 27100 Pavia, Italy; Department of Biomedical Sciences, Humanitas University, 20072 Pieve Emanuele, Milano, Italy; Department of Advanced Biomedical Sciences, Federico II University, 80138 Naples, Italy; Cardiothoracovascular Department, Azienda Sanitaria Universitaria Giuliano Isontina and University of Trieste, 34128 Trieste, Italy; Department of Advanced Biomedical Sciences, Federico II University, 80138 Naples, Italy; Division of Cardiology, Department of Pharmacy, Health and Nutritional Sciences, University of Calabria (UNICAL), 87036 Cosenza, Italy; Advanced Cardiovascular Imaging Unit, Clinica Villa dei Fiori, Acerra, 80011 Naples, Italy

**Keywords:** survey, cardiovascular magnetic resonance, challenges, barriers, competency-based cardiac imaging

## Abstract

**Aims:**

Challenges related to the use of cardiovascular magnetic resonance (CMR) remain a key issue to secure its full clinical impact. This survey aimed to assess the awareness of CMR clinical utility and to collect data on its local usage levels, operational barriers, and report efficacy, with the goal of identifying key obstacles to its effective implementation across Italy.

**Methods and results:**

The CMR Working Group of the Italian Society of Cardiology promoted an online survey targeting Italian physicians involved in direct care of patients with cardiovascular disease. The questionnaire was completed by 709 physicians, mostly working in public or university hospitals (75%); 27% were medical residents. Cardiomyopathies and myocarditis were identified as the most established clinical indications for CMR. 79% of respondents perceived underutilisation of CMR in their local settings, with waiting times exceeding 3 months in 42% of cases. Public hospitals were reported as the primary providers of CMR services (41%), with the majority of CMR reports signed exclusively by radiologists. Obstacles in obtaining clinically useful and effective CMR exams were frequent, with 69% of respondents often encountering issues. Need for an expert second opinion was reported by 27% of participants either often or always. Stress CMR was reported of limited access or unavailable by 79% of respondents.

**Conclusion:**

CMR is highly regarded for its clinical utility but underutilized due to operational barriers, mainly long waiting times and lack of specific competence. Perceived inadequacy in report quality is common and contributes to a consistent rate of second-opinion requests.

## Graphical Abstract

**Graphical Abstract:**
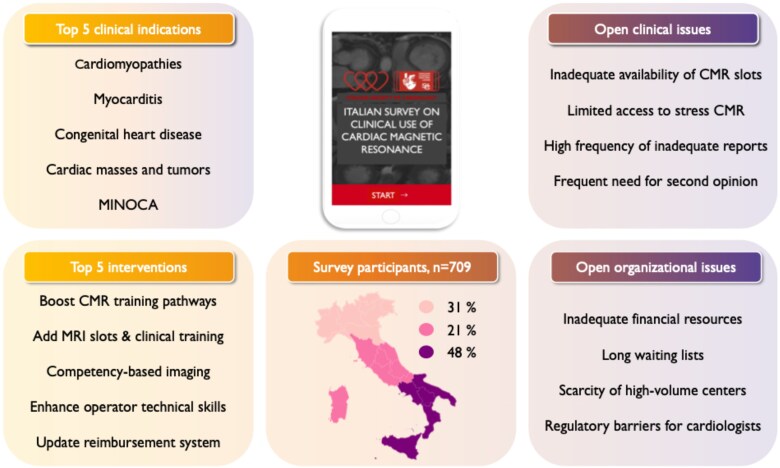

## Introduction

Cardiovascular magnetic resonance (CMR) has emerged as a pivotal imaging tool in the diagnosis and management of a wide spectrum of cardiovascular diseases, providing unparalleled insights into cardiac structure, function, and tissue characterization. During the years and after the production of great amounts of scientific data, the modality has gained full recognition for its diagnostic precision and clinical value in managing a wide range of cardiovascular conditions. Thus, contemporary guidelines strongly advocate for CMR in clinical practice, emphasising its role in precise disease characterization and contribution to improving patient outcomes through early detection, risk stratification and targeted management.^1^ Unfortunately, the adoption and accessibility of CMR remain inconsistent globally and geographically variable,^2,3^ with disparities particularly evident across Europe.^4^ To prevent dramatic consequences on the efficacy of management strategies for patients with cardiac diseases, the gap with current guidelines needs to be considered carefully by the scientific community as well as the institutional and regulatory bodies to promote wider availability and clinical application of CMR.

Operational barriers such as long waiting times and limited availability of dedicated scanners and of high-volume CMR centres may contribute to the underutilisation of CMR across various healthcare settings.^2,5^ Additionally, variability in training and expertise of CMR operators, together with the complexities in managing multidisciplinary collaboration between cardiologists and radiologists can affect the ability to locally provide a CMR service with appropriate timing and quality.^6^

The CMR Working Group of the Italian Society of Cardiology (SIC) conducted a national survey aimed at capturing real-world data on the clinical usage, accessibility, and effectiveness of CMR across Italy. This survey, targeting clinicians directly involved in the care of cardiovascular patients, sought to provide a comprehensive assessment of the current state of CMR application across different regional areas and practice settings. By including responses from a diverse group of healthcare professionals, the survey also aimed to recognize relevant barriers limiting the full clinical implementation of CMR. The proposed questionnaire was designed to highlight the key obstacles preventing CMR from reaching its full potential in Italy, with the goal of informing policy changes and strategic initiatives to improve access to CMR imaging and the overall quality of CMR services.

## Methods

A national online survey on the utilisation of CMR in clinical practice in Italy was led by the SIC CMR Working Group. Physicians involved in the clinical management of patients with cardiac diseases were granted the opportunity to participate in this survey via an accessible web link, which was disseminated through e-campaigns, group e-mails, publications on social media and on the official SIC website. The survey was conducted using a secured online platform accessible via smartphone, laptop, or tablet from October 31st to 28 November 2023. All respondents gave their consent to participate in the study when accessing the online survey.

### Survey design and structure

The survey was administered in Italian and consisted of 20 closed-ended questions utilising, when appropriate, a five-point Likert scale ranging from ‘completely agree’ to ‘completely disagree’ to assess the degree of alignment with various statements (see *Supplementary material* for the full questionnaire). The survey included different sections designed to capture: (i) an overview of the professional profiles of participating physicians; (ii) participants’ perspectives on the clinical utility and usage of CMR; (iii) predominant operational setting, in the local environment, providing CMR services; (iv) insights from respondents into current barriers that may hinder optimal CMR utilisation; (v) considered relevance of holding adequate cardiological competences for CMR operators; (vi) perception of the impact quality of CMR reports may have on clinical risk and professional risk; (vii) consideration for potential modalities of intervention suggested to enhance CMR clinical utilisation.

## Statistical analysis

Descriptive statistics were employed to analyze the survey responses, summarising the distribution of answers across various categorical variables. The results were expressed in terms of percentages, highlighting the frequency of responses for each question. No inferential statistical comparisons were made between subgroups or variables. All statistical analyses were performed using Wizard 2.0 (version 2.0.16) for macOS.

## Results

### Survey participant profile

A total of 709 physicians actively involved in managing patients with known or suspected cardiovascular disease participated in the survey (see *Supplementary material* for the full results report). Among the respondents, 41% had been practising cardiology for over ten years, while 27% were still in training. Of the participants, 46% were employed in public hospital facilities, 29% in university hospitals and 15% in accredited private hospitals. The remaining participants worked in public or private outpatient territorial services. Survey respondents were distributed across various regions of Italy, with Southern Italy accounting for the largest share (48% of participants).

### Utility and use of CMR in clinical management

Regarding the perceived utility of CMR, 96% of respondents recognized CMR as an extremely useful or very useful tool in the management of cardiovascular conditions. The proportion of participants expressing *complete* or *partial agreement* in considering CMR an established clinical indication was very high when assessing cardiomyopathies (100%), myocarditis (99%), cardiac masses/tumors (98%), congenital heart diseases (95%) or MINOCA (93%), but lower when indication is represented by valvular heart diseases (68%), diseases of the thoracic aorta (67%), and acute coronary syndromes other than MINOCA (53%) (*Table 1*). When asked about levels of CMR utilisation in clinical practice, 40% of respondents reported that the modality was *widely underutilized* and 39% considered it *slightly underutilized*. Only 17% believed CMR was appropriately utilized in their settings, with a small percentage (3%) reporting slight or wide overutilisation of CMR.

**Table 1:** Strength of CMR clinical indications as perceived by participants

Clinical indication	Agreement^a^
Cardiomyopathies	100%
Myocarditis	99%
Cardiac masses	98%
Congenital heart disease	95%
MINOCA	93%
Pericardial disease	88%
Ischaemia/Viability	86%
Valvular heart disease	68%
Diseases of the aorta	67%
Acute coronary syndromes	53%

^a^Cumulative proportions of responses as “complete agreement” and “partial agreement”.

### Operational CMR settings

CMR examinations were performed in public hospitals in 41% of cases, in private hospitals in 27% of cases, and in university hospitals in 18% of cases. Regarding reporting practices, report were signed by a radiolgist, with the inclusion of a consultant cardiologist in 27% of cases. CMR reporting done by cardiologists are described as exceptional (5%). 6% of reponders were not aware of the medical speciality of the reporting physician (*Table 2*).

**Table 2:** CMR reporting approaches in Europe and Italy

Reporting physician(s)	ESCR registry^7^	SIRM survey^8^	SIC survey
Cardiologist alone	1.5%	0%	5%
Radiologist and Cardiologist	26.9%	19%	27%
Radiologist alone	71.4%	81%	62%

ESCR, European Society of Radiology; SIC, Italian Society of Cardiology; SIRM, Italian Society of Medical and Interventional Radiology.

### Obstacles and challenges to clinical CMR utilisation

When prescribing a CMR study, clinicians reported to commonly experience obstacles (*often* by 50% and *always* by 19% of respondents) in obtaining the execution of the exam with a timing considered to be appropriate based on clinical considerations. For outpatient CMR services, typical waiting times are reported to exceed 3 months by 42% of respondents and to be <1 month by 8% only. When CMR is prescribed to inpatients, 44% of respondents reported typical waiting times of 4–7 days; inpatients are used to wait 7–30 days according to 29% of responses and <3 days only in 12% of cases (*Table 3*).

**Table 3:** Average waiting time for CMR in outpatients and inpatients

Average waiting time	Percentage frequency (%)
Outpatient setting	
<1 month	8%
1–3 months	39%
3–6 months	28%
6–12 months	14%
Other	12%
Inpatient setting	
<4 days	12%
4–7 days	44%
7–30 days	29%
>30 days	4%
No service	5%
Other	6%

With respect to access to stress CMR, 50% of respondents indicated that the test was not available in their clinical environment, and an additional 29% reported a waiting time to be longer than 3 months from the request. Execution of CMR for patients with cardiac electronic devices, such as ICDs and pacemakers, was reported to be frequently an issue: 20% indicated *limited availability* (3–6 months waiting time), 18% *very limited availability* (6–12 months waiting time) and 16% of respondents stated that CMR was *not available* at all for patients with implanted devices.

**Figure 1:**
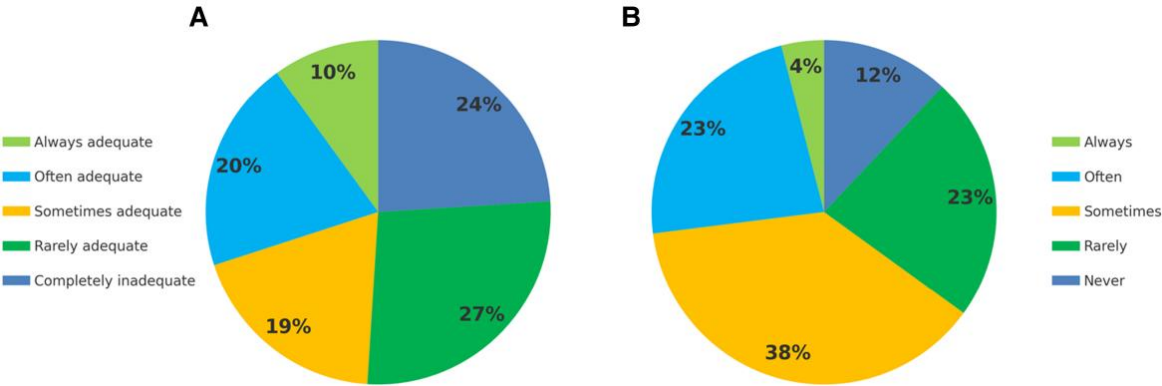
Relevance of adequate cardiological training for CMR operators. Perceived level of involvement of operators with cardiology training (A); frequency of exam re-evaluation or repetition by cardiology-trained operators (B).

Regarding the difficulty in obtaining clinically useful and effective CMR exam, defined as well-suited to the clinical question and providing appropriate and consistent reports, 34% of respondents reported they *often* encountered obstacles, while 32% reported encountering these obstacles *sometimes*. Respondents identified several key obstacles to effective CMR utilisation (*Figure 1*). The most frequently cited barriers included limited availability of dedicated slots for cardiac studies on MRI systems (86% indicating the answers *completely agree* or *partially agree*), long waiting lists (82%), limited availability of high-volume/reference centres for CMR (73%), and limited financial resources allocated by the public health system (71%). Additionally, having operators without adequate cardiology training (58%) and limited access to CMR execution and reporting for non-radiologists (58%) were also reported as relevant barriers.

### Relevance of cardiological training for CMR operators

**Figure 2:**
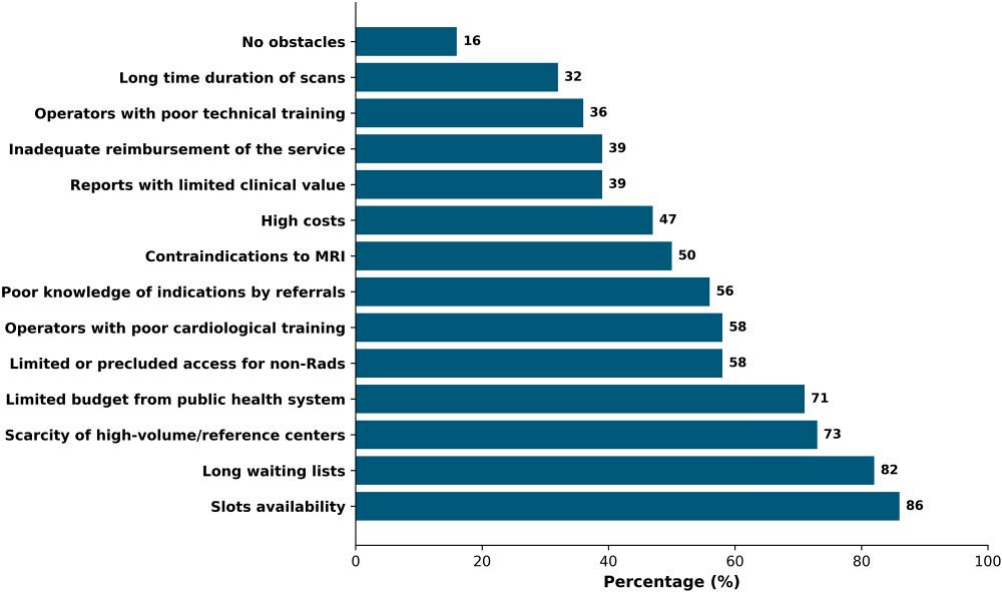
Perceived obstacles to appropriate clinical use of CMR in Italy. The frequency percentages shown in the *x*-axis of the chart represent cumulative proportions of responses, specifically combining both complete agreement and partial agreement. This means that the percentages reflect the total number of respondents who either fully agreed or partially agreed with each barrier being a significant factor affecting CMR access and utilisation. By aggregating these two levels of agreement, the chart provides a clearer picture of the overall perceived importance of each barrier.

There was widespread agreement on the necessity of appropriate cardiological training for an optimal diagnostic performance of CMR, as 83% of respondents *completely agreed* that comprehensive training—including knowledge of ECG, echocardiography, clinical management strategies, and use of drugs for ischaemia assessment—should be considered as essential for optimising CMR practice. Additionally, only 10% of respondents indicated that the involvement of operators with cardiological training in CMR execution and reporting was *always adequate*, while involvement was reported to be *rarely adequate* by 27% and *completely inadequate* by 24% of the participant clinicians (*Figure 2*).

### Impact of CMR report quality on clinical risk and professional risk

Concerns regarding the impact of clinically inadequate CMR reports were highly prevalent among respondents. Eighty-five percent *completely or partially agreed* that inadequate reports (e.g. non-conclusive/informative reports or with incorrect conclusions) can expose patients to significant clinical risk, and 79% *completely or partially* agreed that such reports pose professional and medico-legal risks to physicians. Overall, 90% of respondents agreed that the occurrence of poor-quality reports may negatively affect the efficiency of clinical care pathways as applied to their cardiac patients.

**Figure 3:**
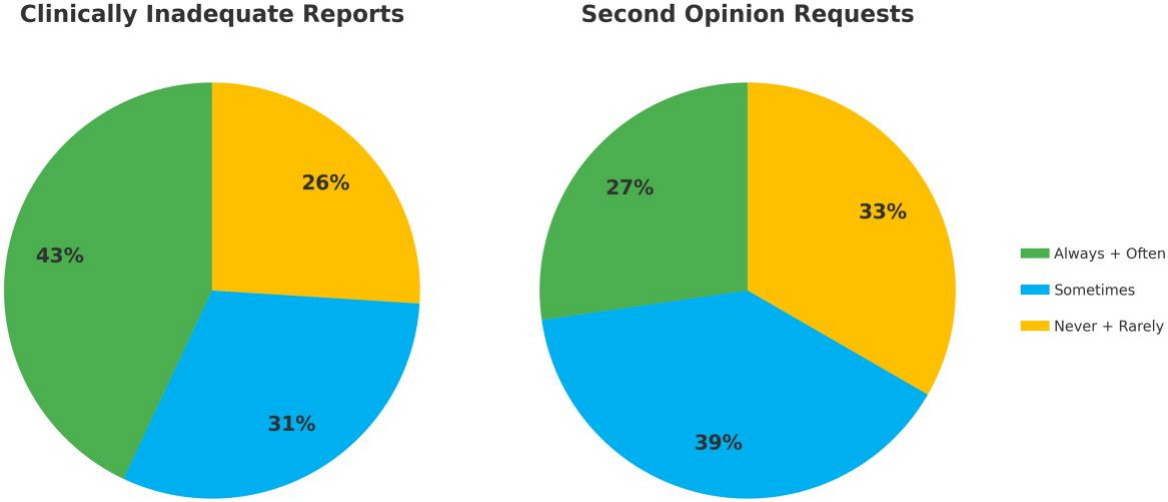
Frequency of clinically inadequate reports and second opinion requests. The diagram on the left illustrates the frequency with which reports are considered clinically inadequate. The diagram on the right shows the frequency of second opinion requests. These visualisations highlight the need for improvement in report accuracy and clinical interpretation to reduce the necessity for second opinions and ensure more effective patient care.

When asked about the frequency of sending their patients for CMR scan re-evaluation or repetition (by operators with cardiological training), 27% of respondents indicated this to be *often* or *always* required in their work environment, while an additional 39% reported that re-evaluation/re-scan was *sometimes* necessary (*Figure 3*).

### Proposed interventions to enhance CMR clinical utilisation

A series of interventions suggested to improve CMR utilisation across clinical settings were all endorsed by participants. These included improvements on technical training (*completely agree* or *partially agree*, 92% of respondents) and clinical training (94%); promotion of educational initiatives targeting clinical referrals (97%); removal of existing regulatory barriers that prevent operators with adequate training, but lacking a radiology specialty diploma, from actively participating in the acquisition and reporting of CMR studies (91%). Additionally, respondents agreed on the necessity to intervene by increasing the number of MRI scanners dedicated to cardiac applications (95%) and of scanning slots available for this type of exam (98%); to update the coding and reimbursement system (86%), by adjusting for complexity, duration, and resources required for CMR exams, and to allocate sufficient budget resources to ensure an adequate number of CMR examinations based on local clinical needs (94%).

## Discussion

This survey conducted by the CMR Working Group of the Italian Society of Cardiology was designed to underscore critical insights into the current state of CMR utilisation in Italy. With responses from 709 healthcare professionals taking care of cardiology patients, the survey captured trends and prevalent challenges in clinical practice, highlighting key structural barriers to CMR accessibility and implementation.

A key takeaway from the survey is the widespread agreement on the clinical utility of CMR, particularly when diagnosing complex cardiovascular conditions such as cardiomyopathies, myocarditis, and congenital heart disease. Almost all respondents recognized CMR role as an essential diagnostic tool. However, despite this recognition, the survey revealed diffuse perception of significant underutilisation of CMR across various clinical settings.

When it comes to the execution and reporting of CMR studies in Italy, the task is predominantly managed by radiologists. Though 30% of participants mentioned that cardiologists are allowed to sign CMR reports in their working environment, these are often co-signed by radiologists. Reports independently produced and signed by cardiologists were reported by only 5% of participants. The shortage of radiologists specialized in cardiac imaging^9^ and the limited volume of their activity contribute to further delays.^8^ These challenges are not unique to Italy; similar issues are evident in other European and non-European countries.

Structural barriers, such as the scarcity of high-volume CMR centres and unequal geographic distribution, contribute significantly to delays in CMR exams. This was evident from the survey, where around 70% of participants reported difficulties in obtaining a CMR exam within a time frame appropriate for the clinical question. Outpatient CMR exams in Italy are often associated with waiting times exceeding 3 months, while for hospitalized patients, approximately one-third reported delays of more than 7 days—considered inadequate for the clinical setting. Delays of this magnitude may hinder the diagnostic and management process of cardiovascular conditions, potentially compromising patient outcomes.

These findings parallel data from the United States, where Medicare data from 2018 revealed similar geographic disparities and delays in CMR access. In the U.S., wait times for stress CMR exams, a key diagnostic modality for ischaemia detection,^8^ ranged from 2 weeks to 3 months depending on the region.^2^ Despite the strong endorsement of stress CMR in clinical guidelines for ischaemia detection,^1,10,11^ over 50% of Italian respondents reported no access to this tool, and those with access often faced delays of over 6 months. In the U.S., as in Italy, the underutilisation of stress CMR is compounded by the limited availability of high-volume centres, further exacerbating access issues. Bridging these gaps and improving access to stress CMR is crucial to ensuring that patients receive timely, guideline-directed care. Expanding access would enable healthcare providers to better manage cardiovascular diseases, improving patient outcomes worldwide.

The majority of participants in this survey testified to a high incidence of barriers to obtaining a CMR study with the appropriate timing and quality of the reports. Of relevance, many regional, local and personal factors may have an impact on a single factor to be considered more or less relevant as compared to others in compromising the accessibility to this imaging modality. Overall, in the perception of physicians taking care of cardiac patients in Italy, principal obstacles to efficient local delivery of CMR studies can be included in two main categories: the first is related to organisational issues, including slots availability, weighting list length and diffusion of high-volume CMR centres; the second category has more to do with a perceived deficiency in clinical perspective when CMR studies are performed and include factors such as the lack of adequate cardiology training for available operators and the persisting regulatory/institutional limitations to an unrestricted access to CMR scanning and reporting for non-radiologists.^7,12^

Our findings echo those of Sierra-Galan et al.^6^ whose global survey across 70 countries pointed to similar challenges limiting access to CMR.^6^ Organisational obstacles—namely scanner availability—and insufficient formal training were identified as major barriers, particularly in developing regions. The global survey also underscored that, regardless of geography, competing imaging technologies and the high cost of CMR remain significant hurdles to its broader implementation. Addressing these challenges will require the expansion of available resources, such as dedicated CMR scanners and appointment slots, as supported by 98% of respondents. Increasing the number of scanners would help alleviate the current bottleneck in CMR access, as supported by 95% of respondents. Dedicated CMR scanners would ensure that cardiology departments can perform timely and specialized imaging, enabling a more efficient workflow and reducing reliance on shared radiology resources. By enhancing the availability of dedicated CMR units, the healthcare system could better meet the growing demand for advanced cardiac imaging, improve patient flow, and shorten waiting times.^13^ Ensuring faster access to CMR could markedly improve patient outcomes, especially in high-risk cases requiring prompt imaging.

Our survey further emphasizes the need for improvements in CMR report quality, with 43% of respondents reporting frequent issues in obtaining clinically useful reports. The frequent need for second opinions—reported by 27% of respondents—indicates variability in report quality, which is often linked to insufficient cardiological involvement in the interpretation process. More than half of the CMR reports are signed exclusively by radiologists, without cardiological consultation in many cases. Addressing this gap through greater interdisciplinary collaboration between radiologists and cardiologists could significantly improve the clinical utility and actionability of CMR reports, in agreement with SCMR, ESCR, and ESR recommendations.^14–16^ Joint interpretation of CMR exams would ensure that reports are not only technically accurate but also clinically relevant, better guiding patient management and optimising resource use *Figure 3*.

The comparison between our cardiology-focused survey and the radiology-driven study by Gatti et al.^8^ highlights key differences in CMR utilisation and reporting practices across Italy. While our survey reported a higher rate of joint radiology-cardiology interpretation (62%) compared with the 18% observed in the radiology study, this suggests that collaboration may be more common in non-academic settings. Both surveys emphasize the need for improved training and competency in CMR interpretation, but our findings stress the importance of cardiology-specific expertise, especially given the critical clinical insights cardiologists contribute to report accuracy and decision-making. Additionally, both studies underline significant challenges in accessing CMR, particularly stress CMR, which remains limited across centres. These results highlight the need for more interdisciplinary collaboration and system-wide efforts to address resource constraints and align practice with clinical guidelines for optimal patient care.

Training remains a crucial area for improvement, with 94% of our respondents advocating for enhanced technical training of CMR operators. While the EACVI has introduced robust certification programs to promote competency,^12^ the survey indicates a persistent gap in the level of cardiology-specific training in CMR interpretation. Enhancing training pathways for radiologists, particularly in the areas of ECG interpretation, ischaemia assessment, and clinical management, would contribute to more accurate and actionable CMR reports. Furthermore, removing regulatory barriers that currently prevent adequately trained non-radiologists from participating in CMR acquisition and reporting would maximize the available expertise within the healthcare system.

The survey also underscores the need for an updated coding and reimbursement structure within the Italian National Health Service. Ninety percent of respondents agreed that the current reimbursement system does not adequately reflect the complexity, time, and resources required for CMR exams, eventually discouraging institutions from expanding their CMR services. A revised reimbursement framework that accounts for the duration and resource intensity of CMR exams could incentivize healthcare providers to increase CMR availability and reduce waiting lists.^17^

## Study limitations

The survey saw greater participation from the southern regions of Italy compared with the central and north, which does not precisely reflect the actual population distribution. This discrepancy may have been influenced by a heightened perception among southern cardiologists of the challenges in accessing CMR, given that healthcare infrastructure in these regions tends to be less robust and distributed compared with the north. This regional imbalance could potentially skew the results, particularly in relation to report waiting times and access to advanced diagnostic tools such as stress CMR. It is plausible that cardiologists in the south, who may face more frequent barriers to CMR access, were more motivated to participate in the survey, thus amplifying certain challenges that might be less prominent in other regions.

Additionally, 25% of the respondents were in-training cardiologists, which may have influenced the findings, particularly regarding perceived obstacles to CMR access. Cardiologists in training often face limitations in gaining hands-on experience with advanced imaging modalities like CMR, which could heighten their perception of access barriers. While this sub-group might have provided valuable insights into training gaps, their impressions could significantly deviate from the experiences of fully qualified cardiologists. However, the survey did include a majority (75%) of participants who are practising cardiologists, primarily working in public hospitals, and a separate analysis of the responses from trainees vs. fully qualified cardiologists did not reveal significant differences in their views on CMR access.

Furthermore, the reliance on a self-reported survey introduces the potential for response bias. Participants who are more engaged or concerned with the issues surrounding CMR may have been more likely to complete the survey, which could lead to an over-representation of certain challenges. This inherent limitation of survey-based research should be considered when interpreting the results, as the responses may not fully capture the experiences of all cardiologists across the country. Prospective registries or complementary large-scale, multicenter evaluations will be necessary to validate these findings and may offer greater alignment with real-world practice, accounting for the limitations of self-reported data.

Lastly, the survey documents the perspective of clinicians and not of radiologists, who play a central role in execution and interpretation of CMR exams. The participation of radiologists in the survey would have been beneficial in identifying, from their perspective, what the cultural barriers are, particularly in terms of specific training and shared reporting. Regarding the presence of technical and structural barriers that limit access to CMR, their contribution will be essential to overcoming these challenges, alongside strategic health policy choices.

## Conclusions

The survey results emphasize a clear lack of equitable access to CMR across Italy and the urgent need for collective efforts to address the barriers hindering the effective and fair use of CMR in clinical practice. Recognising the extensive benefits of CMR for cardiovascular diagnostics and patient management, immediate action is required to integrate greater CMR access within the national health system, coupled with improvements in report quality through enhanced competency of the reporting physicians, or enhancing radiology-cardiology collaboration. Strengthening interdisciplinary cooperation, providing comprehensive cardiology training to practitioners and enhancing the quality of diagnostic reports by reducing this divide is—first and foremost—in the direct interest of our patients. Overcoming this segregation and providing equitable access to CMR for both cardiologists and radiologists will be critical for the advancement of CMR, in Italy and beyond. Strengthening interdisciplinary cooperation and providing comprehensive cardiology training to practitioners are essential to elevate the quality and pertinence of CMR reports. Moreover, our survey findings highlight a strong need for concerted actions finalized to a broader access to stress CMR, which currently remains beyond the reach of most Italian cardiologists and patients. The optimal solution lies in joint educational initiatives for both cardiologists and radiologists, promoting the development of specific competencies in CMR. By embracing a more patient-centric model of competence development and interdisciplinary collaboration, we can bridge current gaps, leading to improved diagnostic accuracy, enhanced clinical outcomes, and more equitable access to high-quality cardiovascular care.

## Supplementary Material

qyaf046_Supplementary_Data

## Data Availability

The authors confirm that the data supporting the findings of this study are available within the article and its Supplementary material. Raw data that support the findings of this study are available from the corresponding author, upon reasonable request.

## Lead author biography

Dr Lorenzo Monti is graduated in Cardiology and Radiology. He has been heading the CMR unit of IRCCS Humanitas Research Hospital in Rozzano (Milan, Italy) for 20 years, and he is actually chief of the Radiology Department at Istituti Clinici Scientifici Maugeri IRCCS, Pavia (Italy). He has gained significant experience in adult cardiac magnetic resonance imaging, personally reporting more than 20 000 CMR scans. His research interest mainly focuses on adult cardiac magnetic resonance, with a special focus on stress CMR and cardiomyopathies.
