# Supplementary material for: Current trends and challenges in the clinical use of cardiovascular magnetic resonance: a survey from the Italian Society of Cardiology
Source: Eur Heart J Imaging Methods Pract. 2025 Apr 17;3(1):qyaf046. doi: 10.1093/ehjimp/qyaf046 (PMC12062520; doi:10.1093/ehjimp/qyaf046)

Supplementary Material

1. **Survey Cover, 1**
2. **Survey Questionnaire, 2-6**
3. **Survey Results, 7-27**

Clinical use of Cardiac Magnetic Resonance in Italy

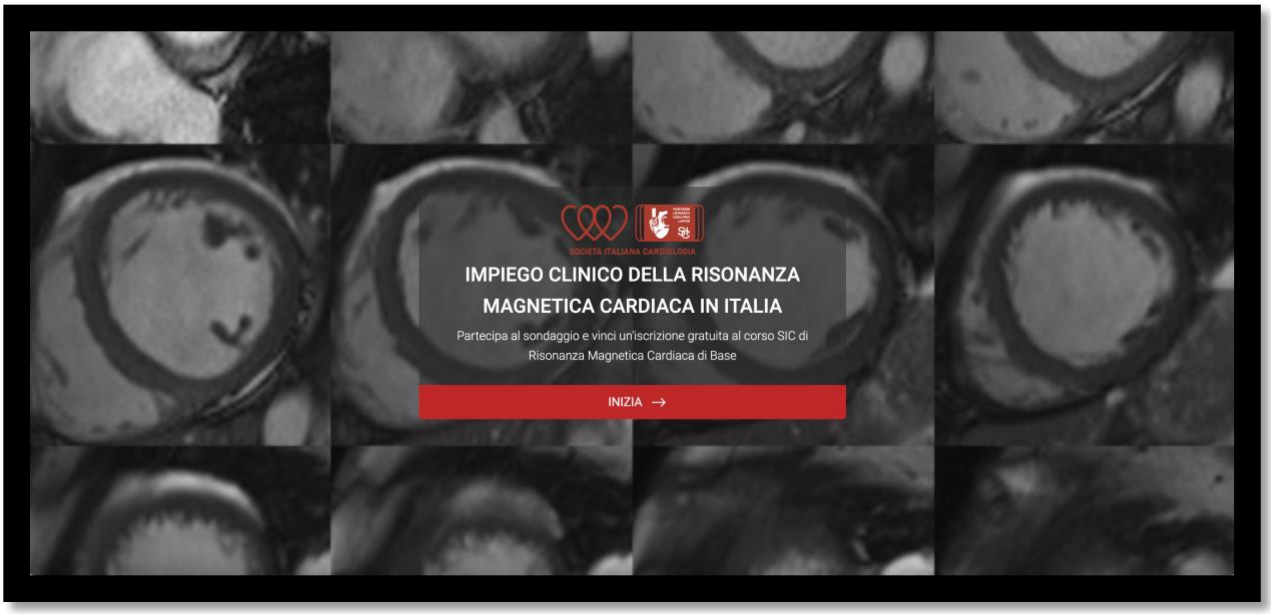

Objectives

The purpose of the survey is to capture and describe the perception of physicians involved in the clinical management of cardiac patients regarding the utility of Cardiac Magnetic Resonance (CMR) and the potential obstacles to its clinical use in Italy. The survey is promoted by the Italian Society of Cardiology (SIC).

Survey Participants

The survey is aimed at all physicians engaged in the clinical management of cardiac patients in Italy (both in hospital and community settings).

1. What is the main operational area of your current clinical activity?

- Public hospital facility
- Accredited private hospital facility
- University hospital facility
- Public outpatient territorial service
- Accredited private outpatient territorial service
- Not active (e.g., retirement, sabbatical year, etc.)
- Other (type text)

2. What is your current status regarding specialist training?

- Physician in training (including participants in specialty schools and PhD programs)
- Physician with completed specialist training < 10 years ago
- Physician with completed specialist training 10 - 20 years ago
- Physician with completed specialist training > 20 years ago
- Other (type text)

3. In which Italian region do you primarily conduct your clinical activity?

Abruzzo
Basilicata
Calabria
Campania
Emilia-Romagna
Friuli-Venezia Giulia
Lazio
Liguria
Lombardia
Marche
Molise
Piemonte
Puglia
Sardegna
Sicilia
Toscana
Trentino-Alto Adige
Umbria
Valle d'Aosta
Veneto

4. Generally, to what extent do you find the information provided by Cardiac Magnetic Resonance (CMR) useful in the clinical management of cardiac patients?

- Extremely useful
- Very useful
- Fairly useful
- Not very useful
- Not at all useful
- I don't know

5. The following is an established clinical indication for a CMR examination:
For each of the listed items, express your preference by selecting the individual entry that interests you: *Completely agree; Partially agree; Neutral; Partially disagree; Completely disagree.*

- Cardiomyopathies
- Congenital heart diseases
- Myocarditis
- MINOCA (Myocardial Infarction with Non-Obstructive Coronary Arteries)
- Cardiac masses and tumors
- Acute coronary syndrome
- Chronic coronary syndrome (including studies of myocardial ischemia and viability)
- Pericardial diseases
- Thoracic aorta pathology
- Heart valve diseases

6. Based on current international guideline recommendations, how do you assess the level of clinical use of CMR in your work environment?

- Widely underutilized
- Slightly underutilized
- Appropriately utilized
- Slightly overutilized
- Widely overutilized
- I don't know

7. In your work environment, are CMR examinations more frequently performed in:

- Public hospital facility
- Accredited private hospital facility
- University hospital facility
- Public outpatient diagnostic center
- Accredited private outpatient diagnostic center
- High-volume/reference facility located in another region
- I don't know

8. Which of the following describes the predominant operational setting (reporting physician) for CMR examinations in your work environment?

- Reporting with validation signature exclusively radiological (without cardiological consultation)
- Reporting with validation signature exclusively radiological (with cardiological consultation)
- Reporting with joint radiological and cardiological validation signature
- Reporting with validation signature exclusively cardiological (with radiological consultation)
- Reporting with validation signature exclusively cardiological (without radiological consultation)
- No CMR service is available
- I don't know

9. Generally, how often do you encounter obstacles in obtaining the execution of a CMR with timing appropriate to the clinical question?

- Always
- Often
- Sometimes
- Rarely
- Never

10. In your work environment, what is the average waiting time for a CMR prescribed to an outpatient?

- Less than 1 month
- 1-3 months
- 3-6 months
- 6-12 months
- More than 12 months
- The service is not available
- I don't know

11. In your work environment, what is the average waiting time for a CMR prescribed to a patient during hospitalization?

- Within 3 days
- 4 to 7 days
- 7 to 30 days
- Over 30 days
- The service is not available
- I don't know

12. How do you assess the current availability of pharmacological stress CMR examinations in your work environment?

- This type of examination is not available
- Very limited availability (waiting list between 6 and 12 months)
- Limited availability (waiting list between 3 and 6 months)
- Fairly available (waiting list between 1 and 3 months)
- Available (waiting list under 30 days)
- I don't know

13. How do you assess the current availability of CMR examinations for patients with cardiac electronic devices (ICD, pacemakers, etc.) in your work environment?

- This type of examination is not available
- Very limited availability (waiting list between 6 and 12 months)
- Limited availability (waiting list between 3 and 6 months)
- Fairly available (waiting list between 1 and 3 months)
- Available (waiting list under 30 days)
- I don't know

14. In your work environment, how often do you face challenges in obtaining a clinically useful and effective CMR exam (one that is well-suited to the clinical question and provides appropriate and consistent reports)?

- Always
- Often
- Sometimes
- Rarely
- Never

15. The following condition constitutes a significant obstacle to the appropriate clinical use of CMR in your work environment:
For each of the listed items, express your preference by selecting the individual entry that interests you: *Completely agree; Partially agree; Neutral; Partially disagree; Completely disagree.*

- Poor knowledge of the indications for the examination by clinical cardiologists
- Limited local availability of dedicated slots for heart studies on existing MR systems
- The available operators have poor cardiological training
- The available operators have poor technical training on the operation of MR equipment
- Access to the methodology is limited or denied for non-specialist operators in Radiology
- Long waiting lists
- Limited clinical value of the produced report
- Excessive duration of the individual examination
- High costs
- Limited local availability of high-volume/reference centers
- Inadequate economic reimbursement for the service
- Contraindications to MRI (e.g., claustrophobia, metallic implants, etc.)
- Limited financial resources allocated by the public health system
- The current obstacles are minimal or non-existent

16. To what extent do you believe that appropriate cardiological training (including adequate knowledge of ECG, echocardiography, clinical management strategies, use of drugs for ischemia detection, etc.) of physicians involved in the performance and reporting of CMR examinations is necessary for optimal diagnostic yield of the method?

Express your preference by selecting the individual entry that interests you: *Completely agree; Partially agree; Neutral; Partially disagree; Completely disagree.*

17. Generally, how do you assess the current level of involvement of physicians with cardiological training in the performance and reporting of CMR examinations in your work environment?

- Completely inadequate
- Rarely adequate
- Adequate in some cases
- Often adequate
- Always adequate

18. In your work environment, how often have you needed to have a CMR examination re-evaluated (or repeated) by an operator with adequate cardiological training after it was initially performed at a center with operators of limited cardiological training?

- Always
- Often
- Sometimes
- Rarely
- Never

19. How much do you agree/disagree with the following statements regarding the clinical impact of CMR reports? For each of the listed items, express your preference by selecting the individual entry that interests you: *Completely agree; Partially agree; Neutral; Partially disagree; Completely disagree.*

- The production of inconclusive/informative CMR reports or reports with incorrect conclusions exposes my patients to significant clinical risks.
- The production of inconclusive/informative CMR reports or reports with incorrect conclusions exposes me to significant professional and medicolegal risks.
- The production of inconclusive/informative CMR reports or reports with incorrect conclusions has negative repercussions on the effectiveness and efficiency of clinical care pathways defined within the National Health System.

20. In what measure do you believe the following intervention could contribute to making the use of CMR more efficient and effective in your work environment?

- Increasing the number of MRI machines dedicated to cardiac applications
- Increasing the number of slots available for cardiac studies on existing MRI machines
- Improving the technical training of operators regarding the functioning of MRI equipment
- Improving the clinical training of operators regarding various aspects of cardiac pathology
- Removing regulatory barriers that limit/prevent operators with adequate training/certification from being involved in the acquisition/reporting of CMR examinations, if not holding a specialization diploma in Radiodiagnostics or equivalent
- Promoting training pathways aimed at increasing knowledge of the current indications and clinical applications of CMR
- Updating the coding and reimbursement system of the Italian National Health Service for each examination (taking into account complexity, duration, and resources used)
- Allocating sufficient budget resources to ensure an adequate number of CMR examinations based on local clinical needs

Clinical Use of Cardiovascular Magnetic Resonance in Italy

What is the main operational area of your current clinical activity?

709 Responses

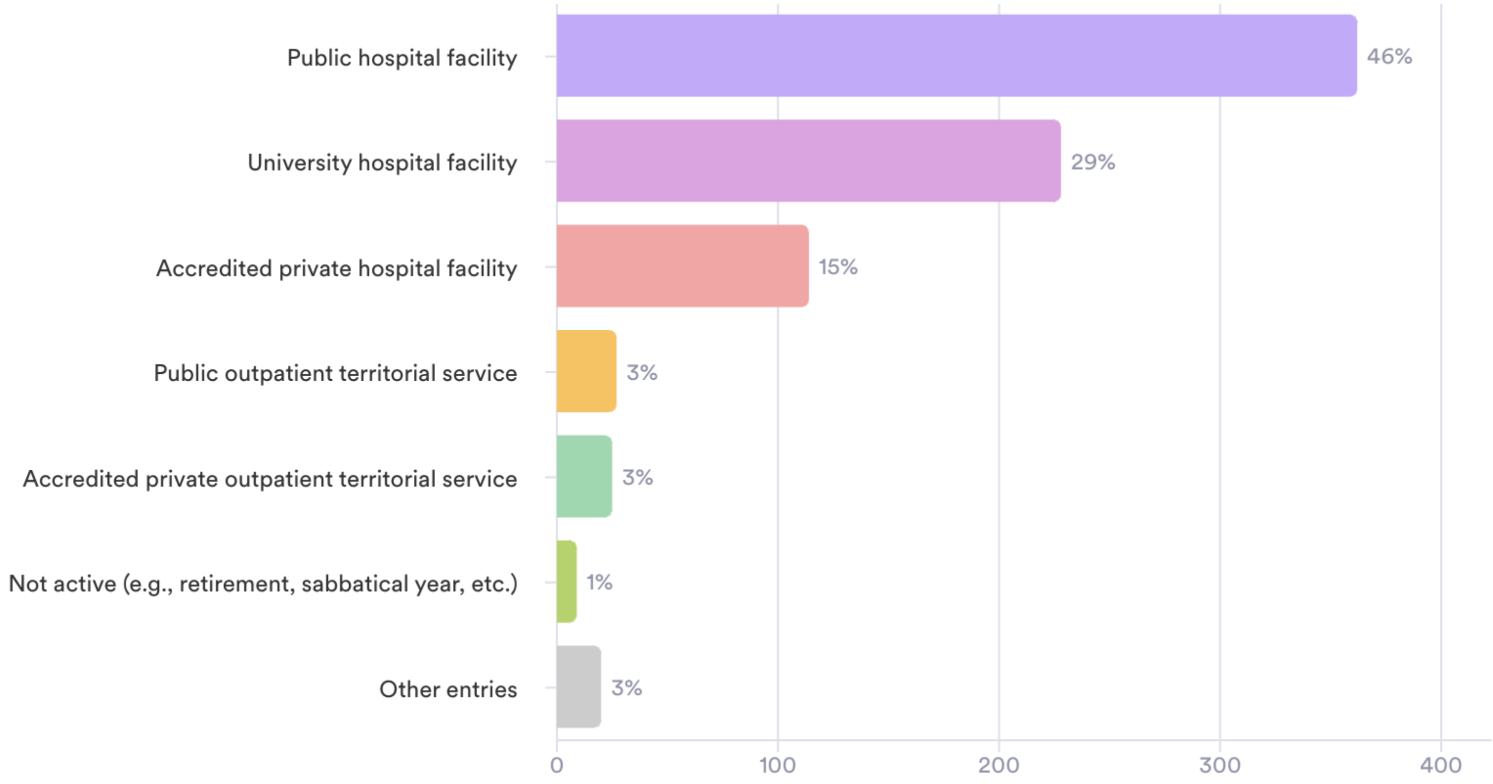

What is your current status regarding specialist training?

709 Responses

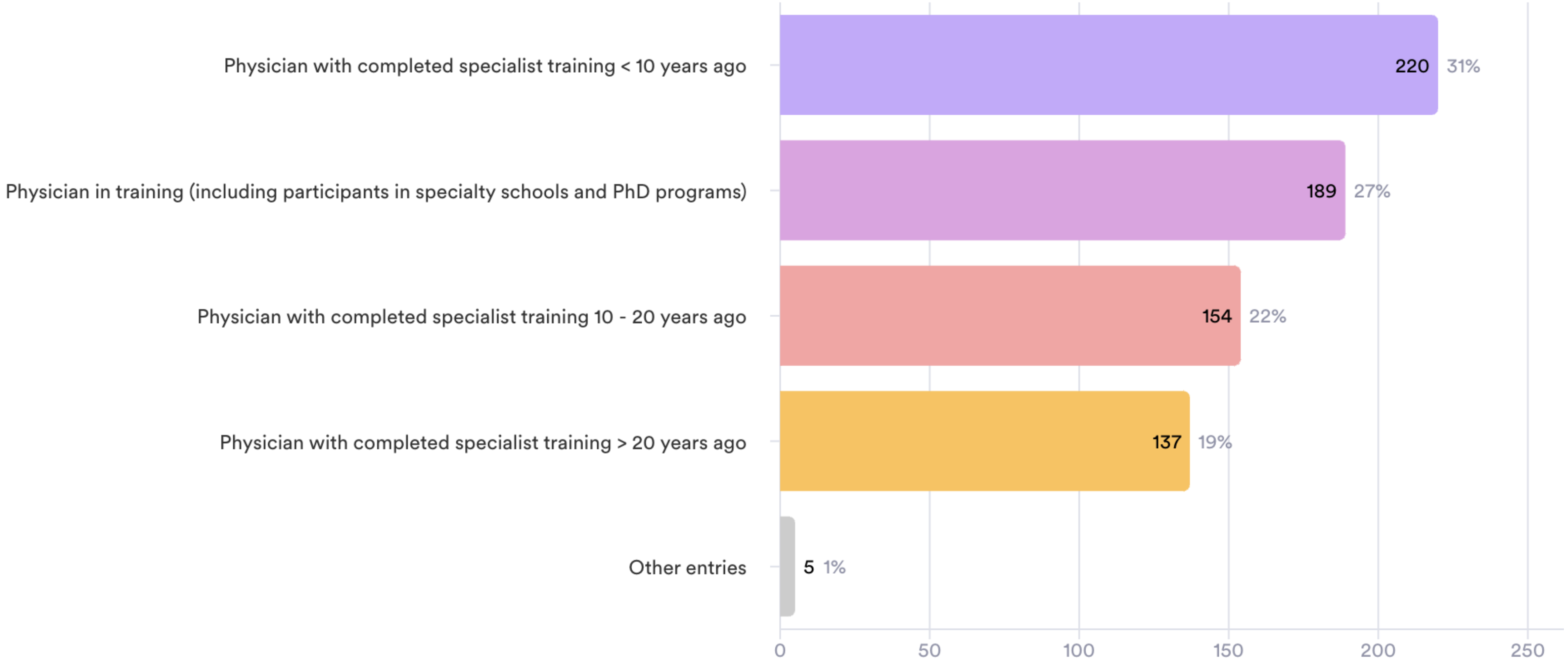

In which Italian region do you primarily conduct your clinical activity?

709 Responses

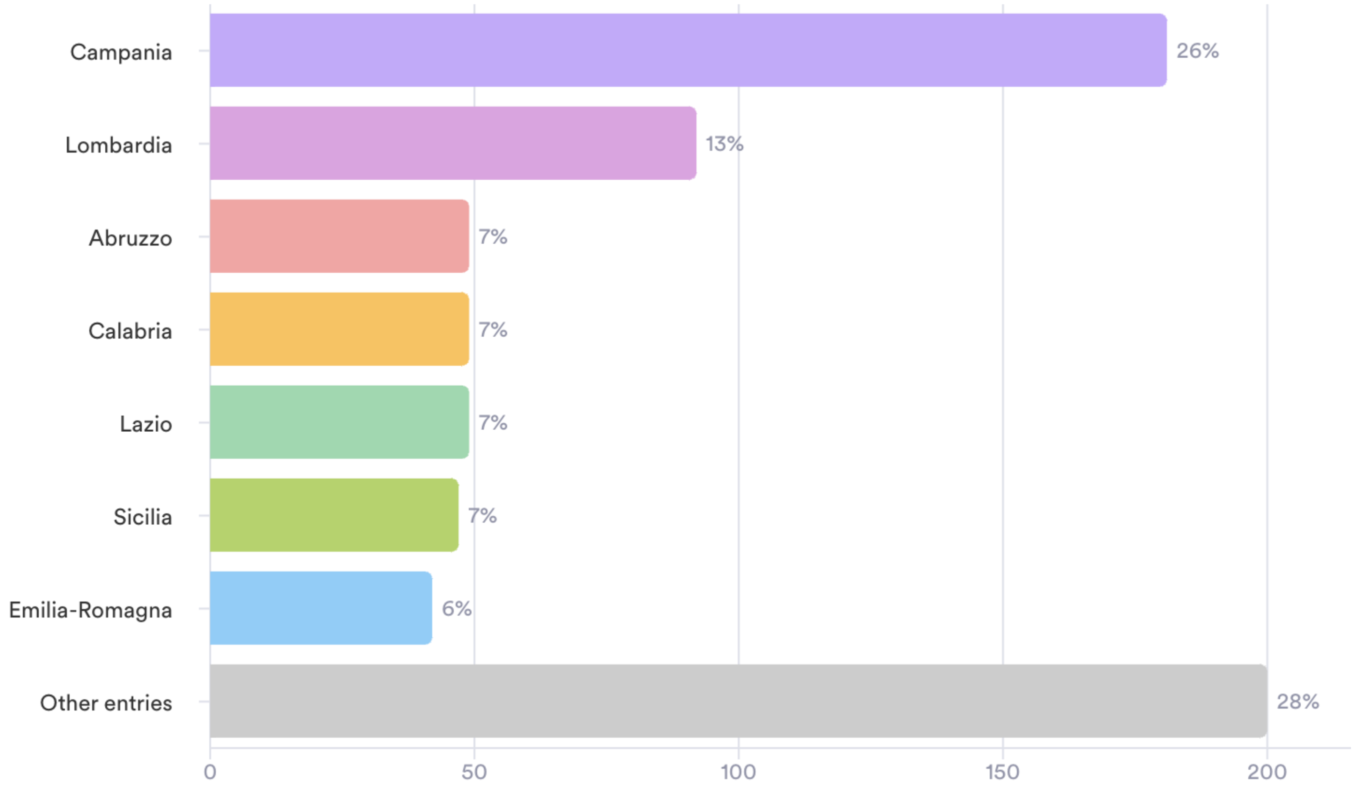

How useful do you find the information provided by CMR imaging in the clinical management of cardiological patients?

709 Responses

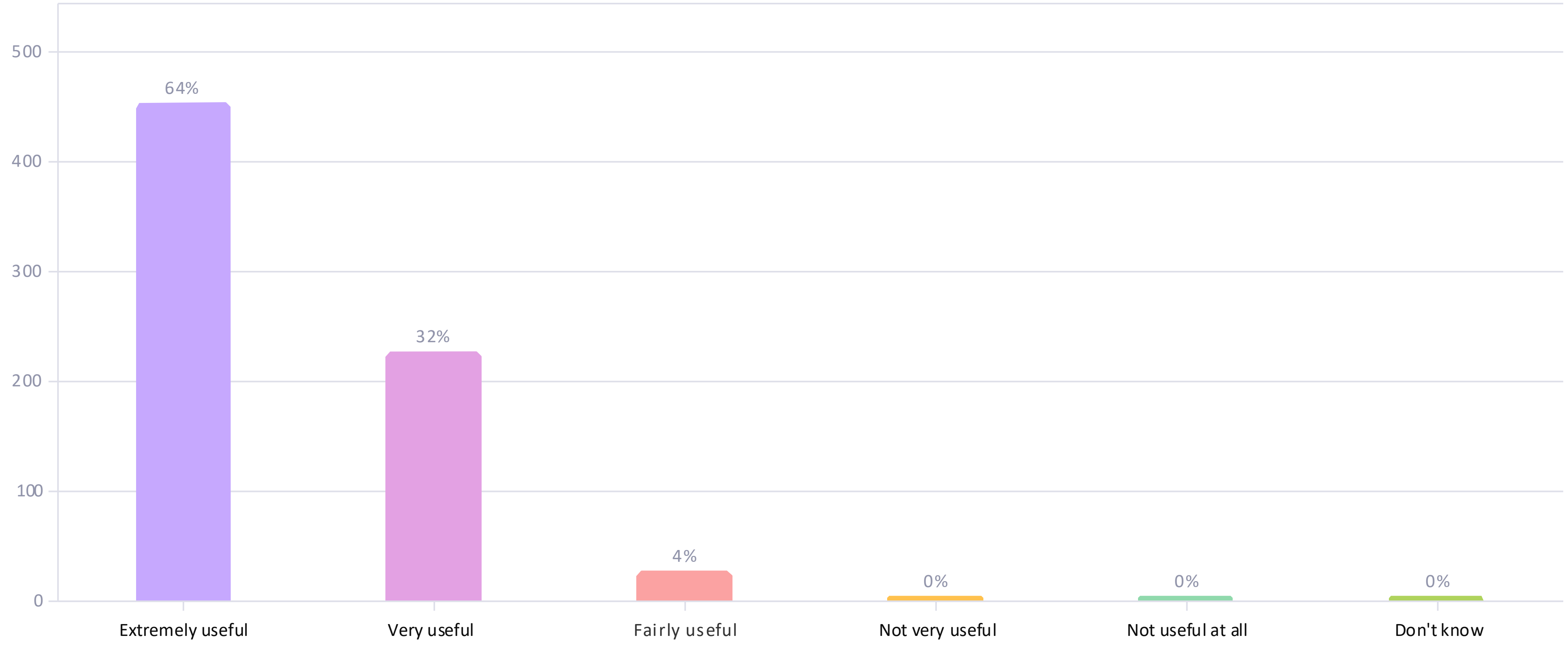

The following is an established clinical indication for a CMR examination

709 Responses

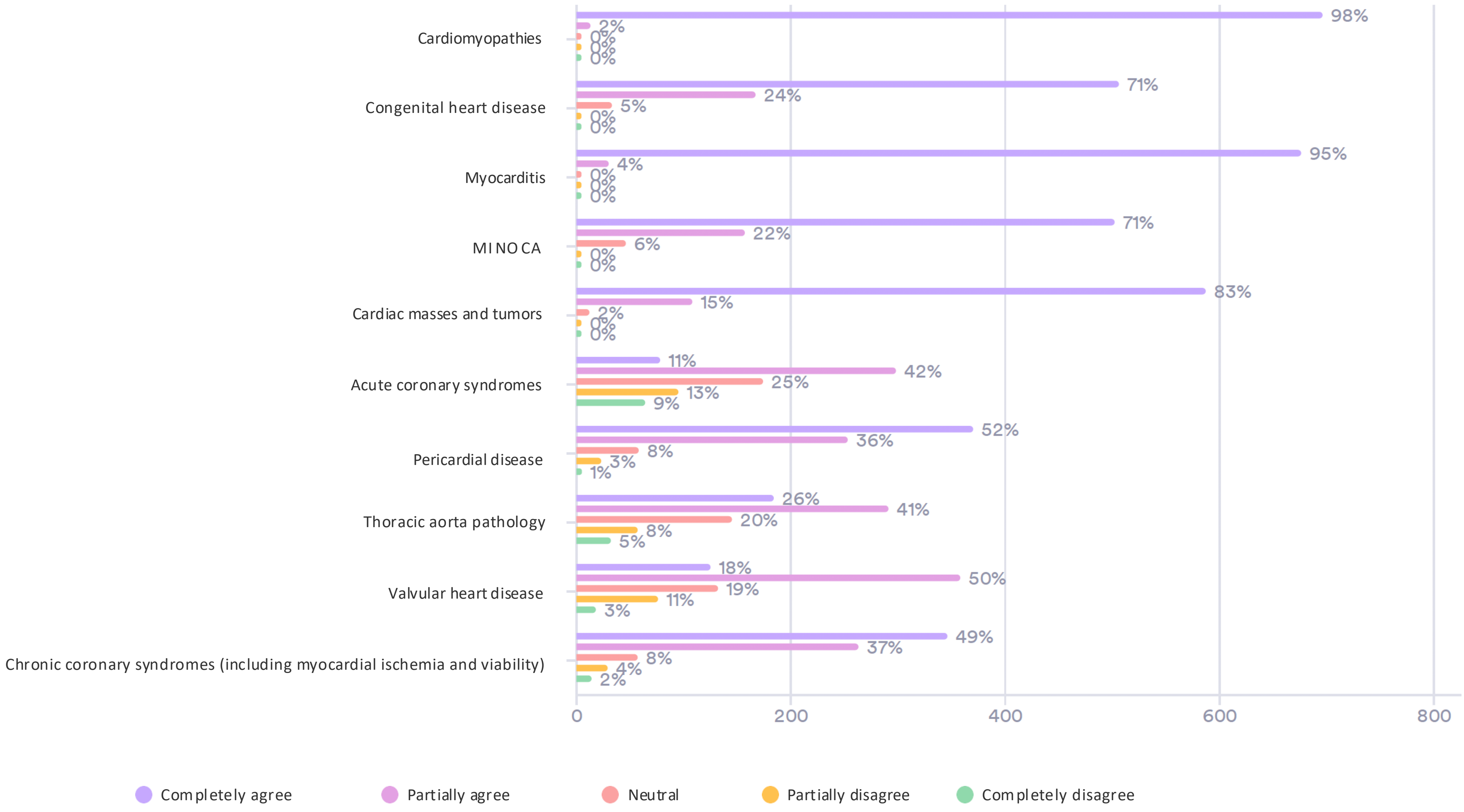

Based on current international guideline recommendations, how do you assess the level of clinical use of CMR in your work environment?

709 Responses

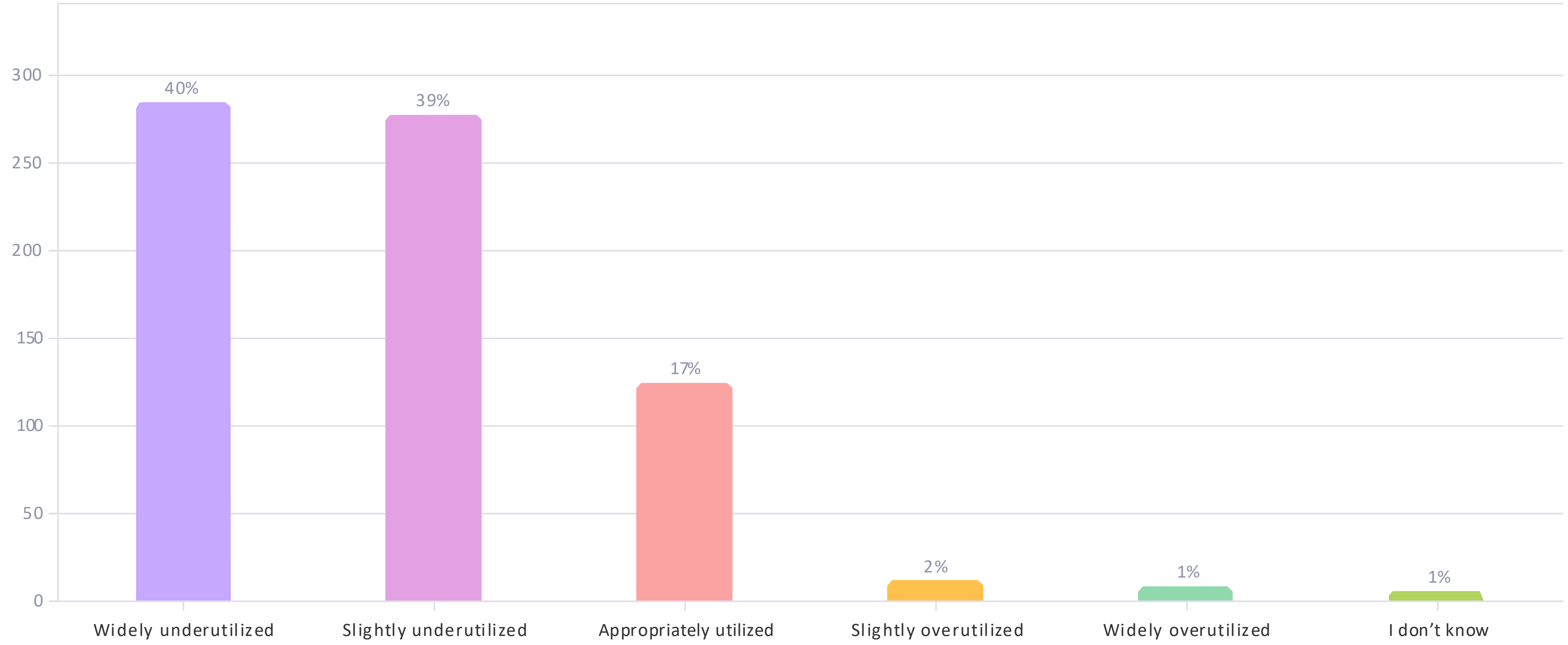

In your work environment, are CMR examinations more frequently performed in:

709 Responses

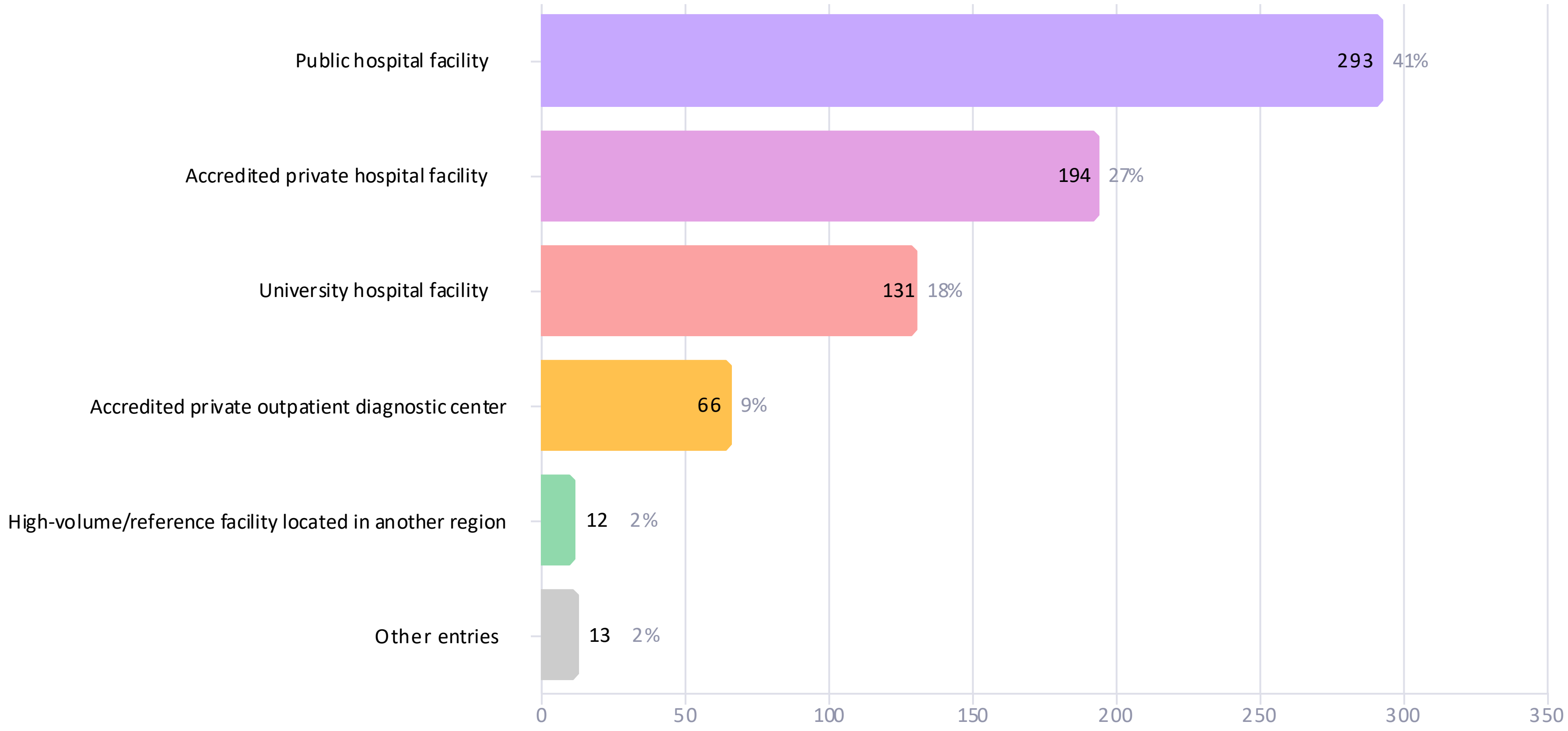

Which of the following describes the predominant operational setting (reporting physician) for CMR examinations in your work environment?

709 Responses

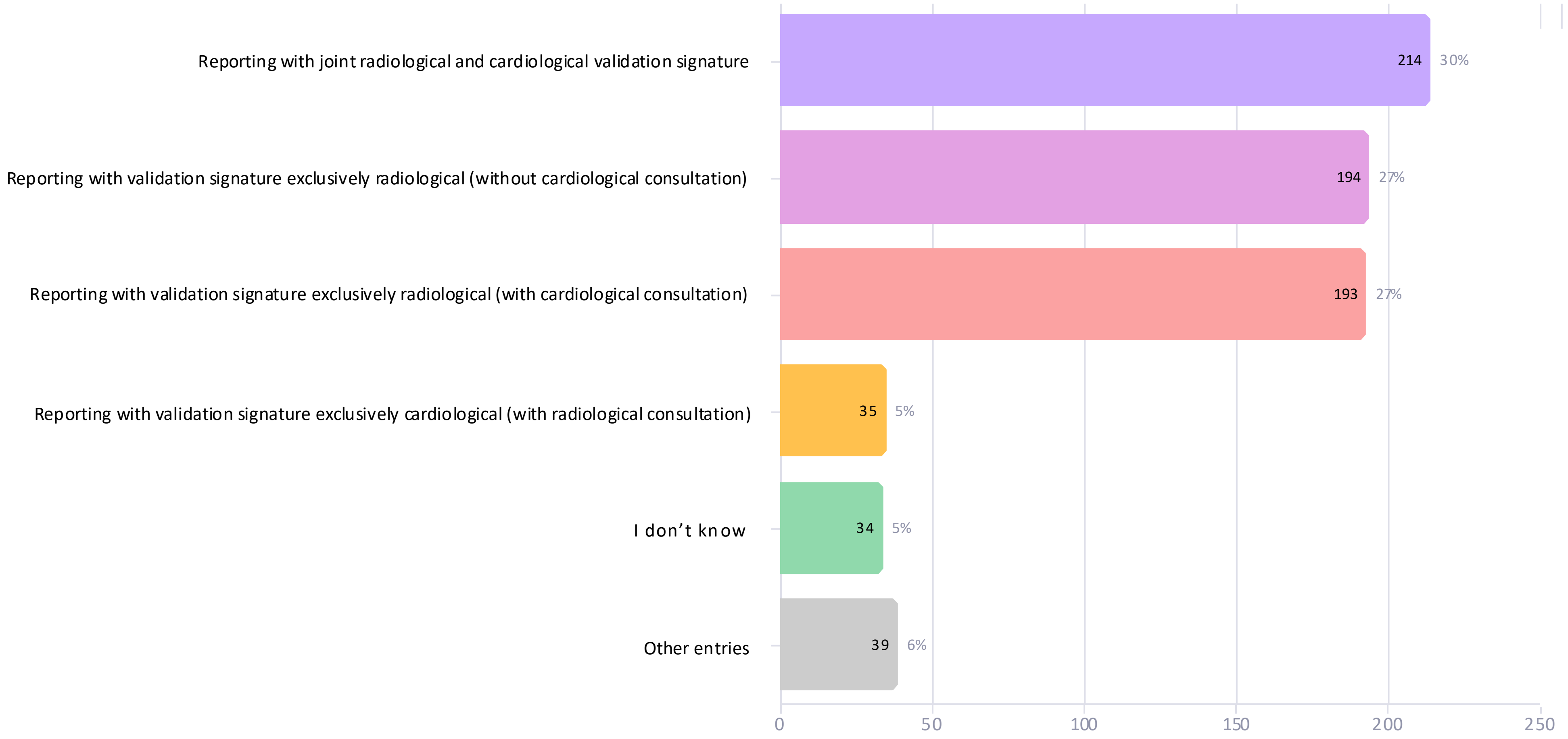

In general, how often do you encounter obstacles in obtaining a CMR with a timing of execution adequate for the clinical question?

709 Responses

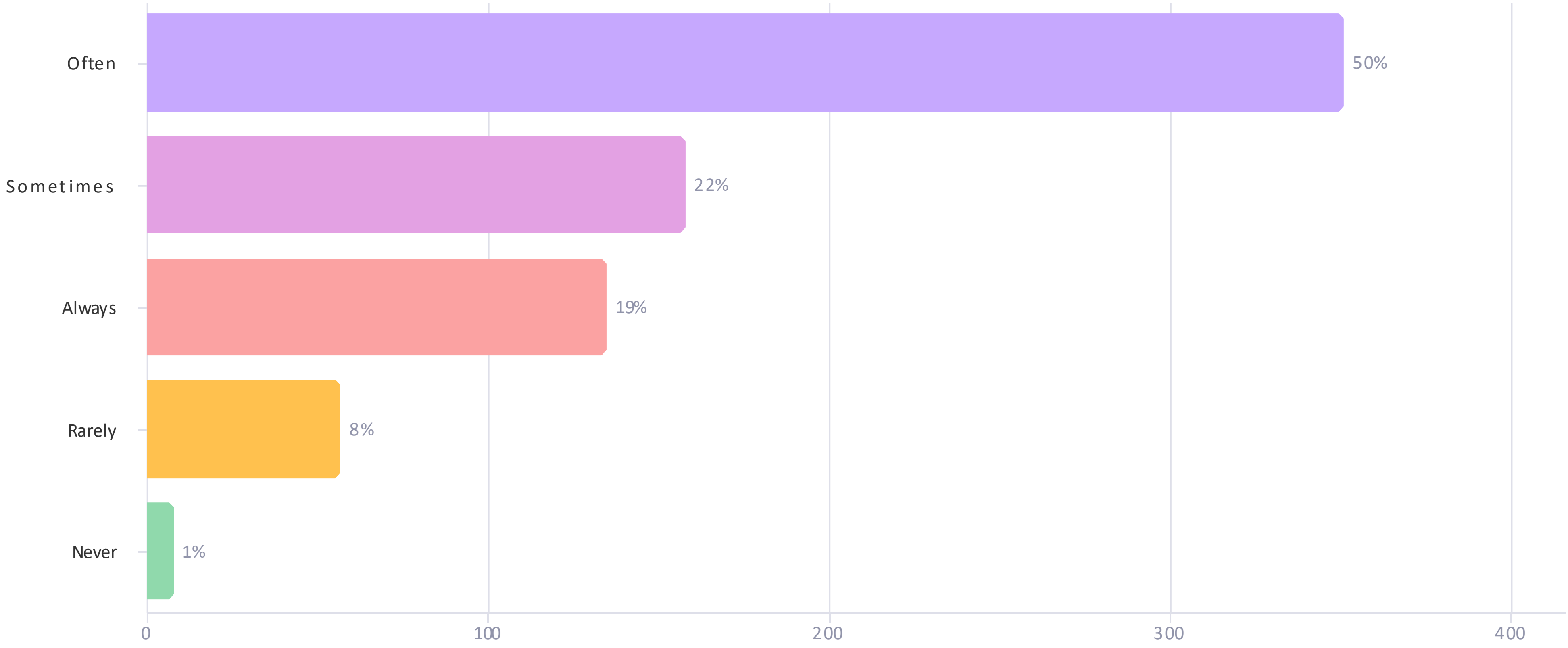

In your work environment, what is the average waiting time for a CMR prescribed to an outpatient?

709 Responses

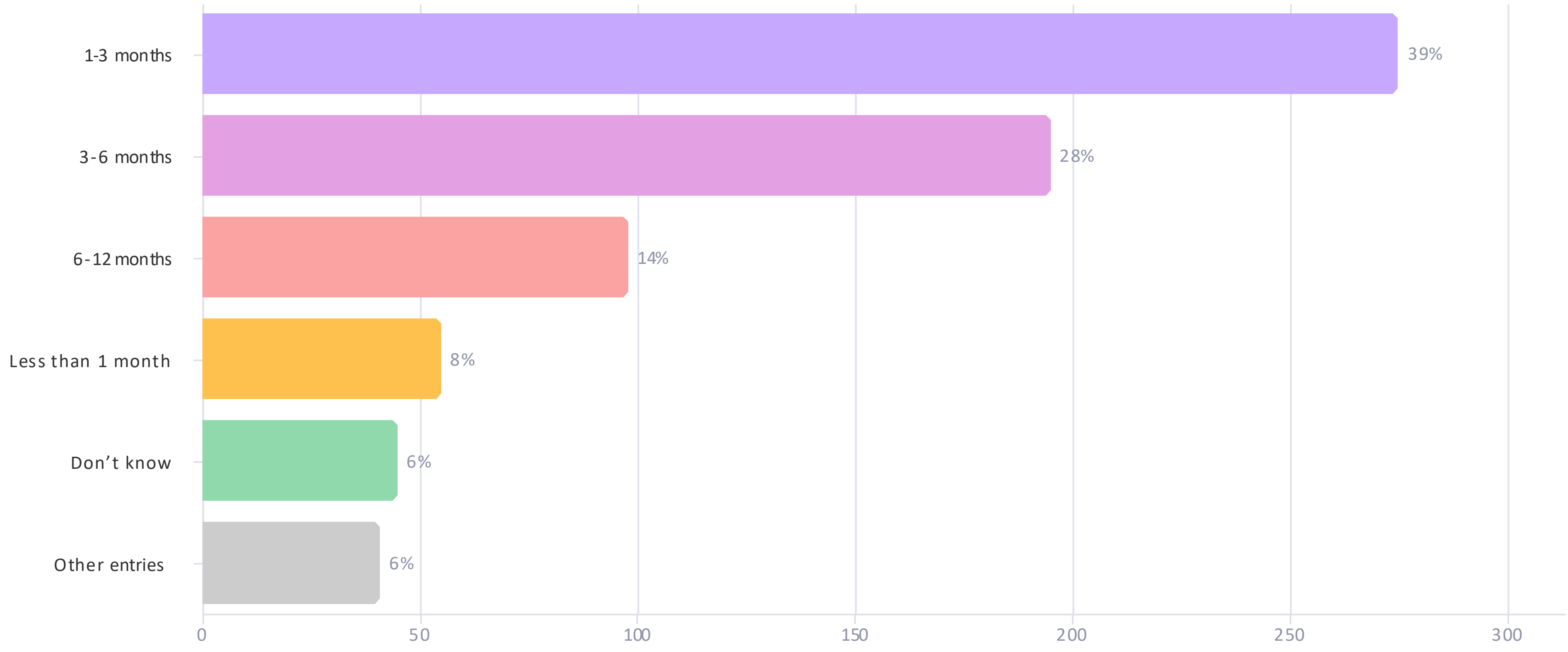

In your work environment, what is the average waiting time for a CMR prescribed to a patient during hospitalization?

709 Responses

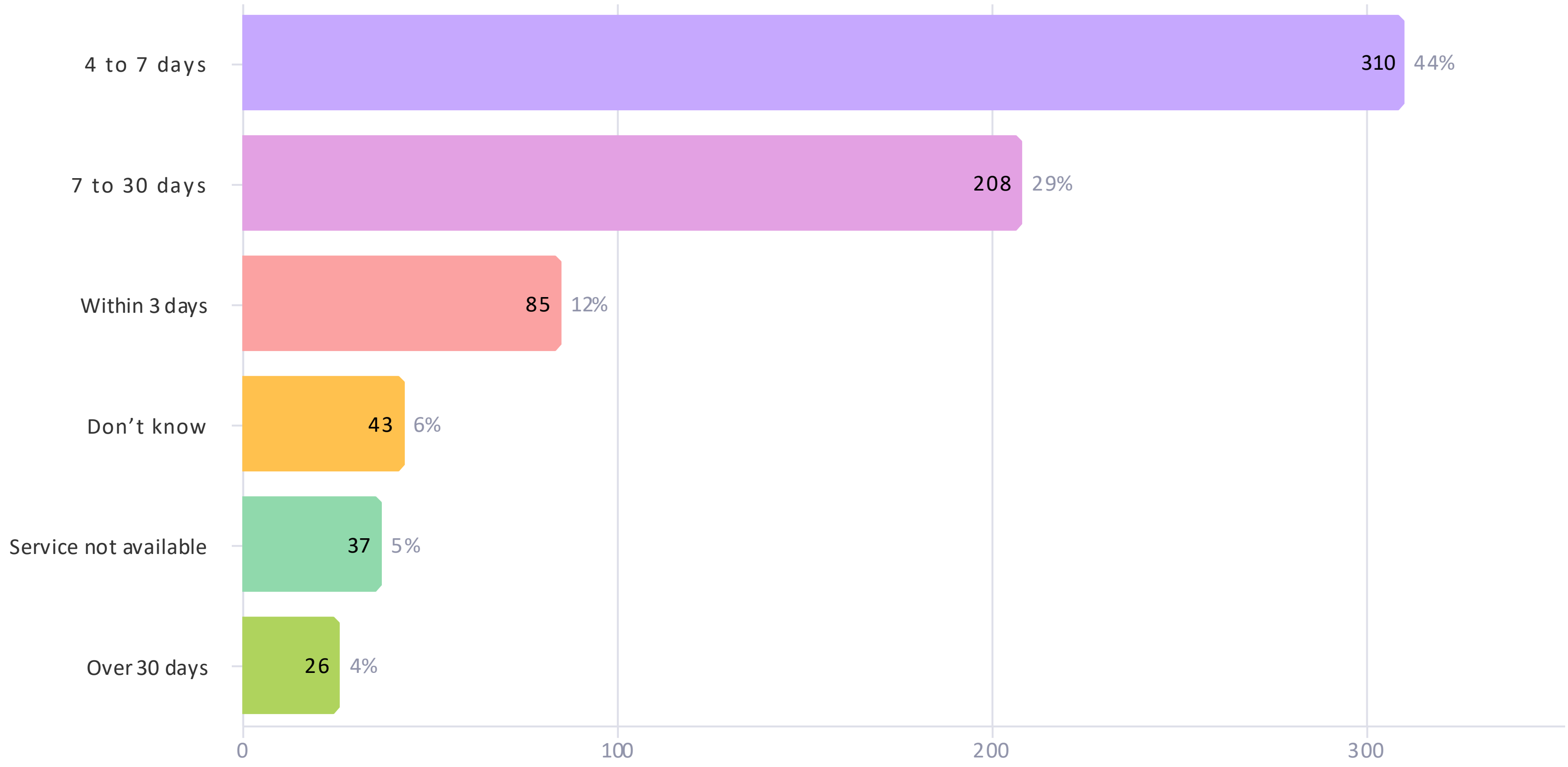

How do you rate the current availability of pharmacological stress CMR exams in your work environment?

709 Responses

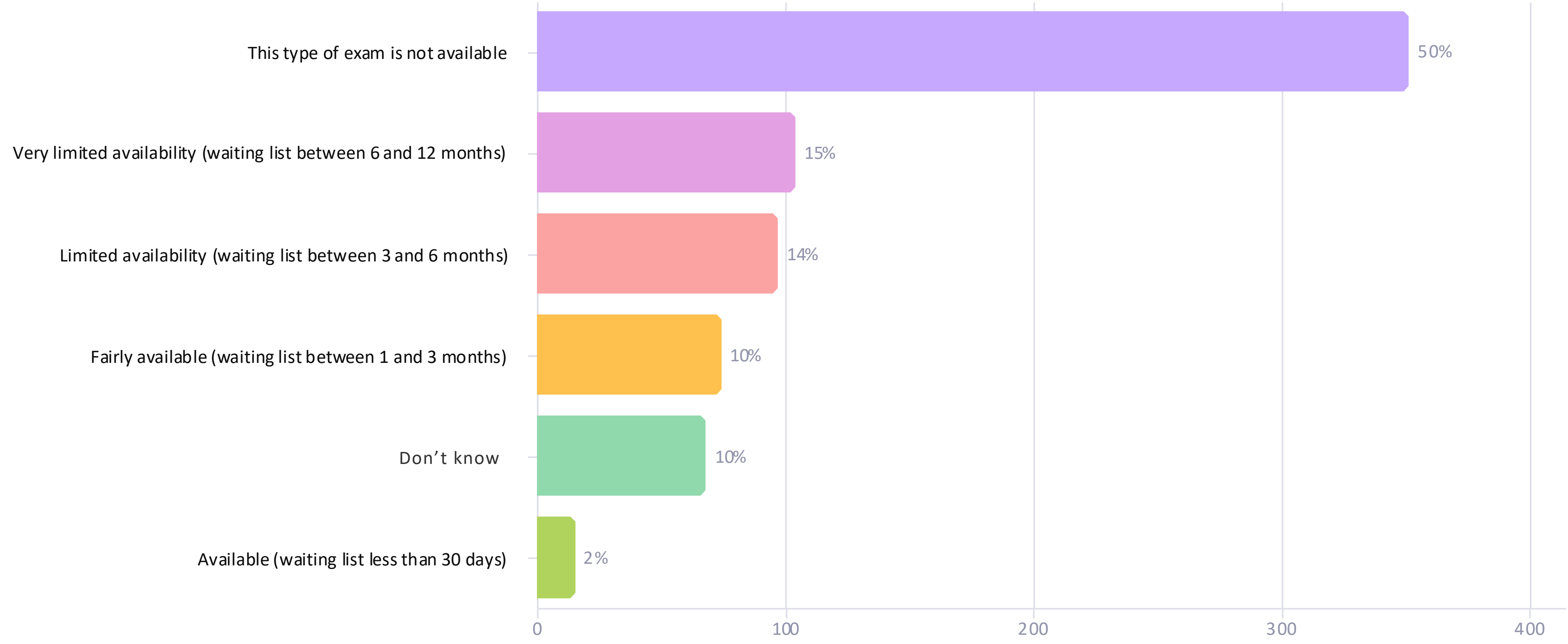

How do you rate the current availability of CMR exams for patients with cardiac electronic devices (ICD pacemakers etc.) in your work environment?

709 Responses

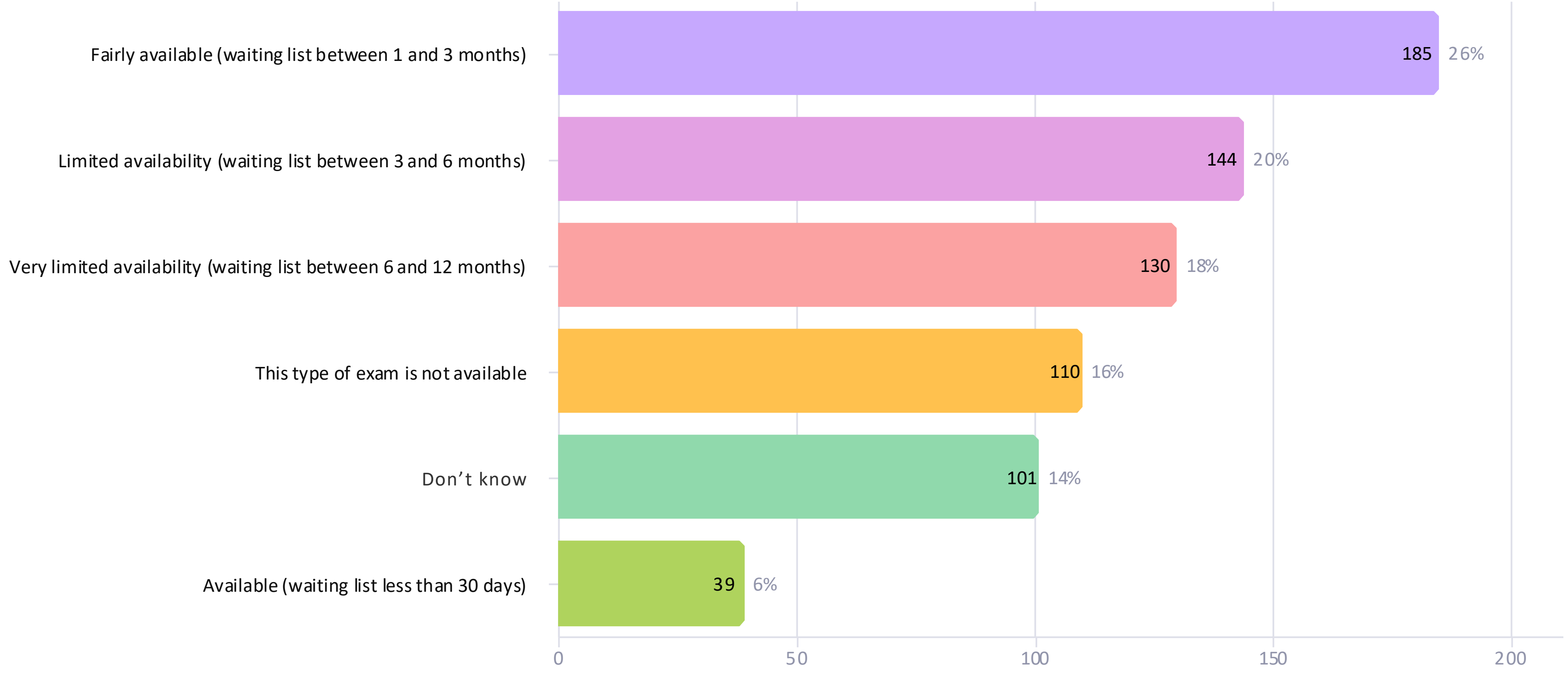

In your work environment, how often do you face challenges in obtaining a clinically useful and effective CMR exam (one that is well-suited to the clinical question and provides appropriate and consistent reports)?

709 Responses

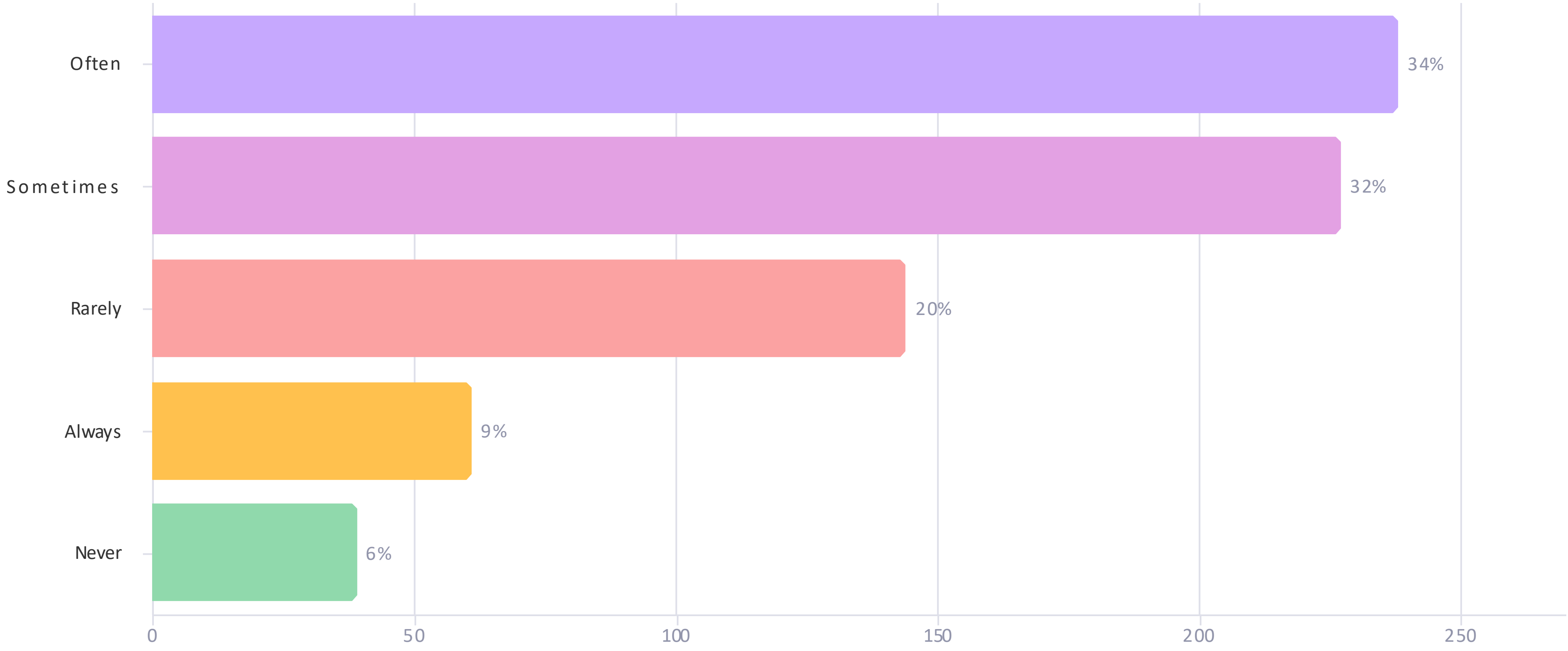

The following condition constitutes a significant obstacle to the appropriate clinical use of CMR in your work environment:

709 Responses

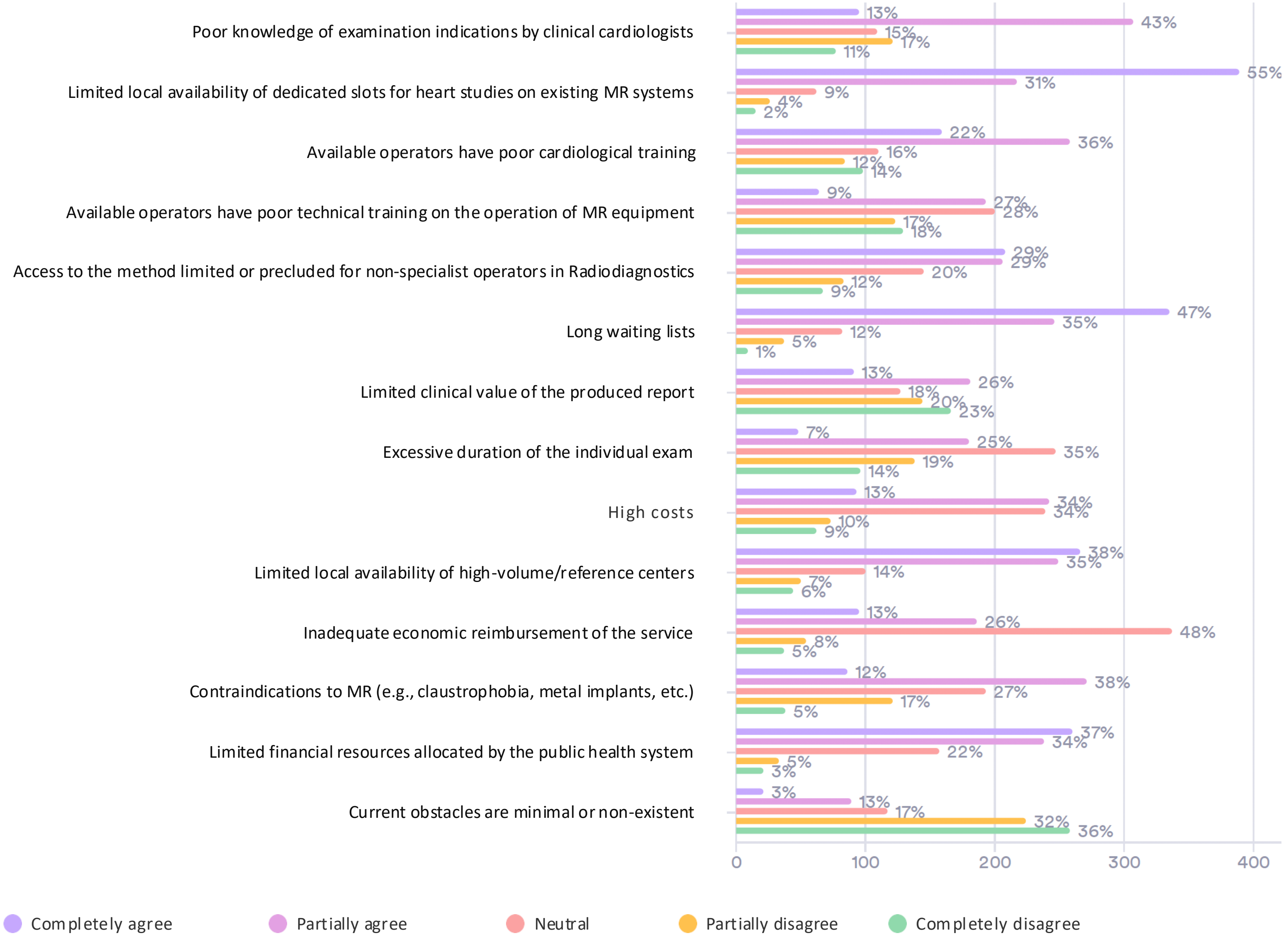

To what extent do you believe that appropriate cardiological training (including adequate knowledge of ECG, echocardiography, clinical management strategies, use of drugs for ischemia assessment, etc.) of physicians engaged in the execution and reporting of CMR exams is necessary for optimal diagnostic performance of the method?

709 Responses

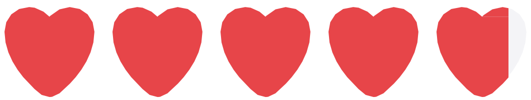

4.75
Avg. Response

709
Responses

Data	Response	%
♥♥♥♥♥	585	83%
♥♥♥♥♡	95	13%
♥♥♥♡♡	15	2%
♥♥♡♡♡	3	0%
♥♡♡♡♡	11	2%

How do you rate the current level of involvement of doctors with cardiological training in the execution and reporting of CMR exams in your work environment?

709 Responses

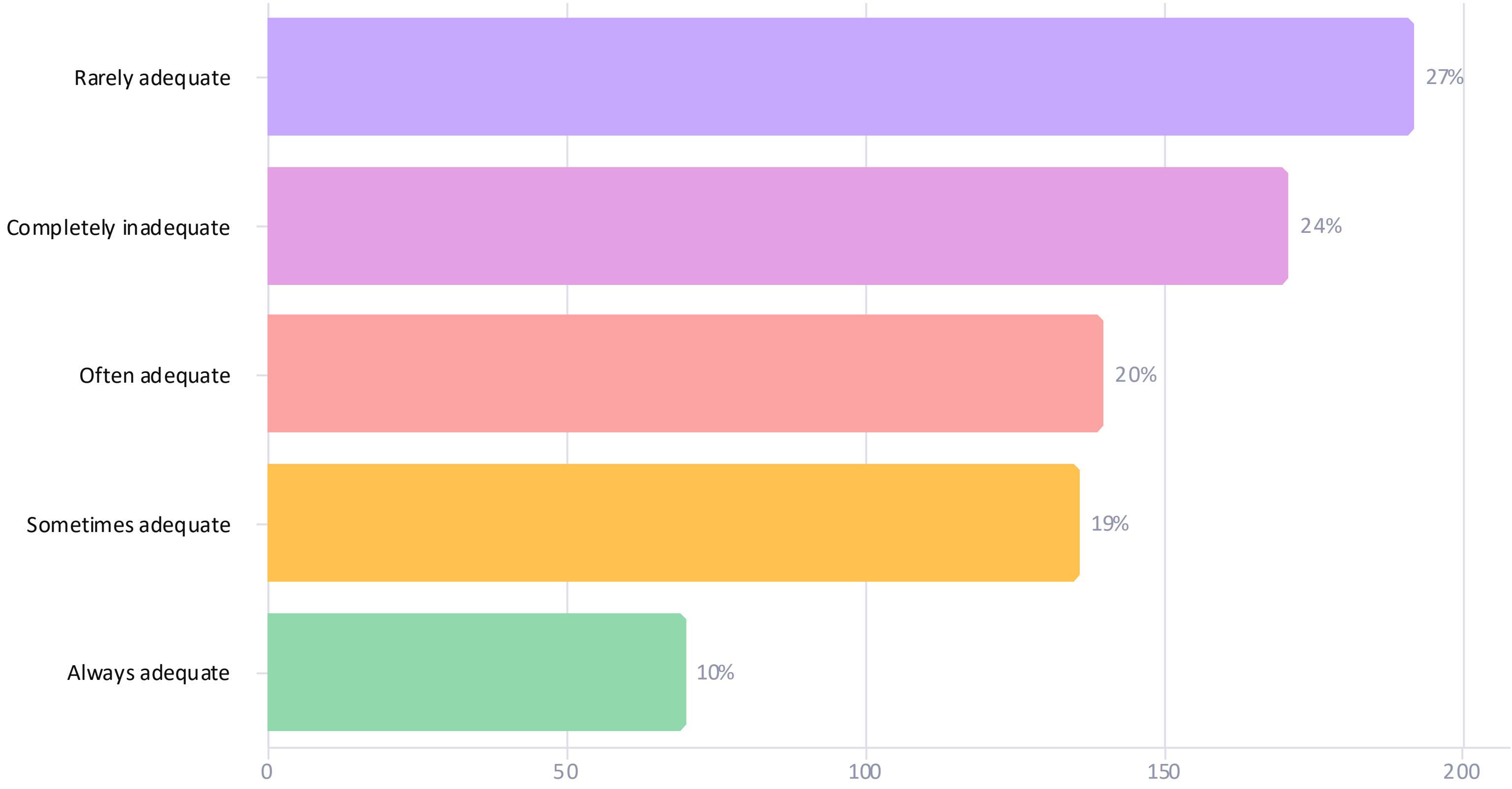

How much do you agree/disagree with the following statements regarding the clinical impact of CMR reports?

709 Responses

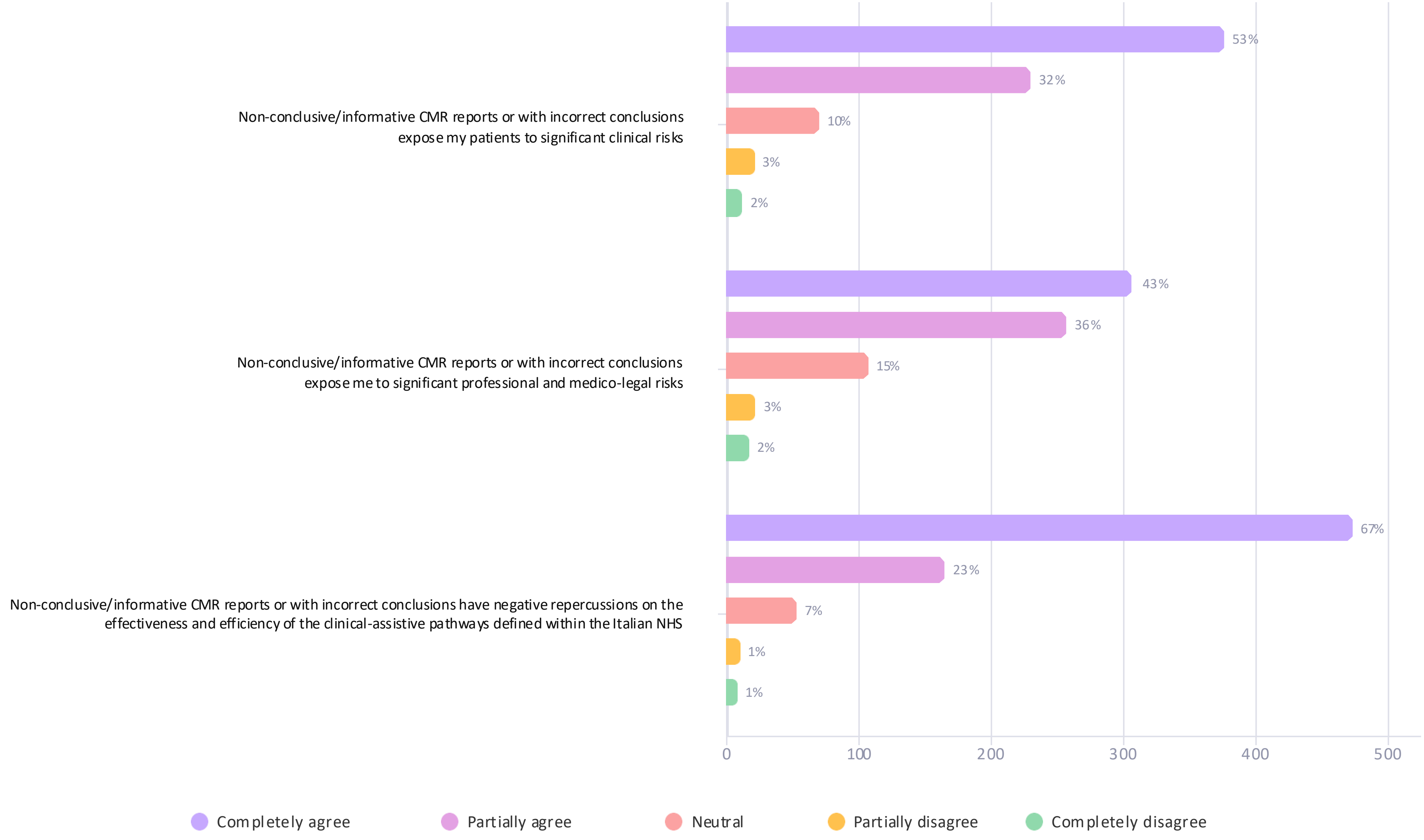

In your work environment, how often have you needed to have a CMR exam re-evaluated (or repeated) by an operator with adequate cardiological training, conducted at a center with operators with limited cardiological training?

709 Responses

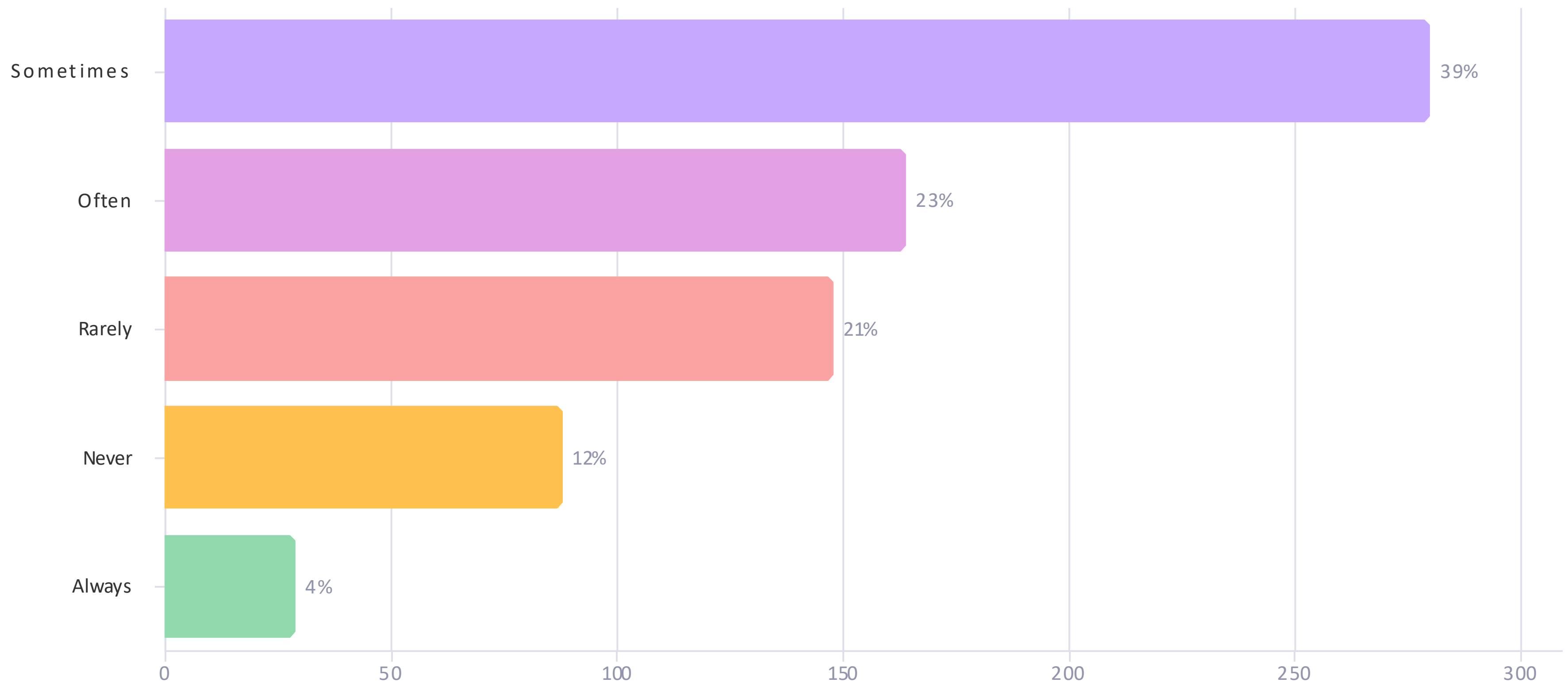

In what measure do you believe the following intervention could contribute to making the use of CMR more efficient and effective in your work environment?

709 Responses

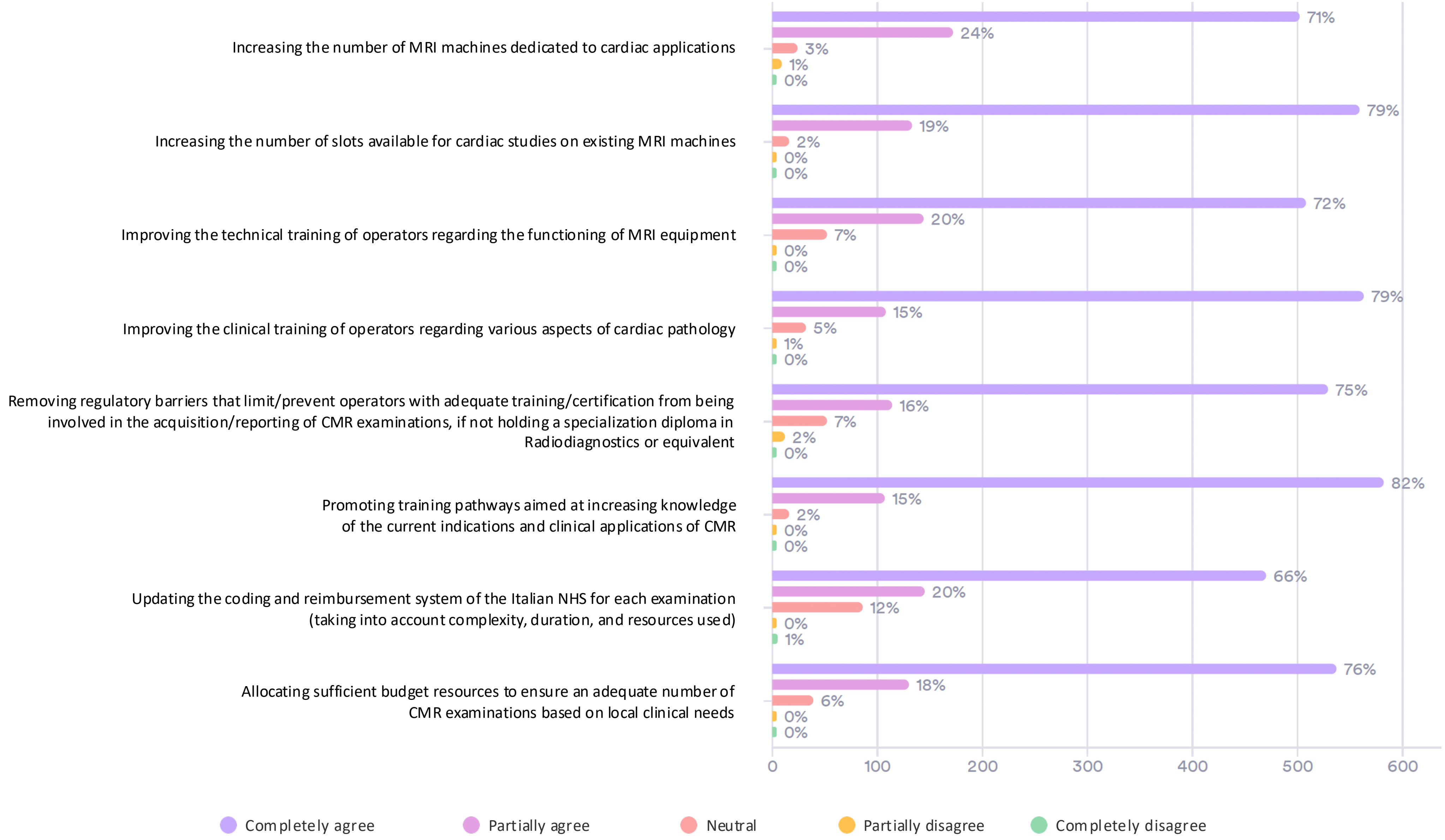

Supplement: qyaf046_Supplementary_Data [file qyaf046_supplementary_data.pdf]
